# The *de novo* DNA methyltransferase 3B is a novel epigenetic regulator of MYC in multiple myeloma, representing a promising therapeutic target to counter relapse

**DOI:** 10.1186/s13046-025-03382-y

**Published:** 2025-04-17

**Authors:** Catharina Muylaert, Lien Ann Van Hemelrijck, Arne Van der Vreken, Robbe Heestermans, Hatice Satilmis, Emma Verheye, Elina Alaterre, Catharina Olsen, Nathan De Beule, Kim De Veirman, Eline Menu, Karin Vanderkerken, Jérôme Moreaux, Elke De Bruyne

**Affiliations:** 1 Translational Oncology Research Center (TORC), Team Hematology and Immunology (HEIM), Vrije Universiteit Brussel, Brussels, Belgium; 2 Translational Oncology Research Center (TORC), Team Hematology and Immunology (HEIM), Vrije Universiteit Brussel, Universitair Ziekenhuis Brussel (UZ Brussel), Brussels, Belgium; 3 Department of Clinical Biology, Vrije Universiteit Brussel (VUB), Universitair Ziekenhuis Brussel (UZ Brussel), Brussels, Belgium; 4 Diag2Tec, Montpellier, France; 5 Clinical Sciences, Research Group Reproduction and Genetics, Centre for Medical Genetics, Vrije Universiteit Brussel (VUB), Universitair Ziekenhuis Brussel (UZ Brussel), Brussels, Belgium; 6 Brussels Interuniversity Genomics High Throughput Core (BRIGHTcore), Vrije Universiteit Brussel (VUB), Université Libre de Bruxelles (ULB), Brussels, Belgium; 7 Interuniversity Institute of Bioinformatics in Brussels (IB)2, Université Libre de Bruxelles (ULB)-Vrije Universiteit Brussel (VUB), Brussels, Belgium; 8 IGH, CNRS, University of Montpellier, Montpellier, France; 9 Laboratory for Monitoring Innovative Therapies, Department of Biological Hematology, CHU Montpellier, Montpellier, France; 10 Institut Universitaire de France, Paris, France

**Keywords:** Multiple myeloma, Epigenetics, DNMT3B, Relapse

## Abstract

**Background:**

The plasma cell malignancy multiple myeloma (MM) remains incurable due to the inevitable development of drug resistance (DR). Epigenetic modifiers are frequently mutated or deregulated in MM patients, contributing to MM progression and relapse. Overexpression of the *de novo* DNA methyltransferase 3B (DNMT3B) in MM has been reported, correlating with poor prognosis. However, its exact role in MM cell biology and relapse remains elusive.

**Methods:**

To evaluate the basal expression and prognostic value of DNMT3B mRNA in terms of overall survival the publicly available gene expression profiling datasets GSE2658, GSE9782, GSE4581, E-MTAB-372, E-TABM-1088 and E-TABM-937 were used. Both the DNMT3B selective inhibitor Nanaomycin A and genetic knockdown using a doxycycline inducible shRNA against DNMT3B were used to target DNMT3B. Viability and apoptosis were assessed using respectively a CellTiter-Glo assay and AnnexinV/7AAD stainings. Cell proliferation was measured by BrdU incorporation and cell cycle analysis, while the clonogenic capacity was evaluated by a colony formation assay. Finally, RNA-seq was performed upon genetic knockdown.

**Results:**

Here, we show that *DNMT3B* is significantly increased in the relapsed setting and high *DNMT3B* levels are strongly correlating with disease progression and high-risk disease, irrespective of the treatment. Targeting DNMT3B using either genetic inhibition or the selective inhibitor Nanaomycin A strongly impaired MM cell growth, survival and clonogenicity. Moreover, Nanaomycin A reduced viability of primary MM cells from newly diagnosed and relapsed patients. Mechanistic studies revealed that DNMT3B inhibition mainly affects cell cycle and stemness-related transcriptional programs. Notably, DNMT3B depletion affected the stability of the master cell cycle regulator MYC, thereby reducing c-MYC levels and cell viability both in parental and c-MYC overexpressing cells. Finally, Nanaomycin A (re)sensitized MM cells to bortezomib, melphalan and anti-CD38 monoclonal antibodies (daratumumab, isatuximab).

**Conclusion:**

Collectively, our findings uncover DNMT3B as a targetable vulnerability in high-risk patients with high DNMT3B/MYC levels.

**Supplementary Information:**

The online version contains supplementary material available at 10.1186/s13046-025-03382-y.

## Introduction

Multiple myeloma (MM) is the second most common hematological malignancy characterized by the accumulation of malignant plasma cells in the bone marrow. Today’s first line treatment typically consists of a triplet regimen of dexamethasone in combination with 2 modern agents, including proteasome inhibitors (PIs, bortezomib and carfilzomib), immunomodulators (IMiDs, lenalidomide and pomalidomide) and monoclonal antibodies (moAbs, daratumumab); followed by autologous stem cell transplantation if patients are eligible [1]. This strategy has significantly increased life expectancy for MM patients by six to ten years. Yet, almost all patients relapse due to the development of drug resistance (DR) and with each round of relapse, the cancer becomes more treatment-resistant. Emerging targeted immunotherapeutics, including chimeric antigen receptor (CAR) T cells and bispecific T-cell engagers, are promising, but the clinical benefit has so far been only incremental as patients are still relapsing. The 5-year overall survival rate remains less than 60%, demonstrating the urgent need to find new therapeutic approaches to prevent relapse and decease [2].

It is now widely recognized that MM is not only a genetic disorder, but also an epigenetic one [3]. In MM, the normal epigenetic landscape is completely disrupted, as evidenced by genome-wide (global) DNA hypomethylation, locus-specific DNA hypermethylation of cancer-associated and/or specific B cell genes, and abnormal expression patterns and/or genetic defects in the epigenetic modifiers (epiplayers) [4, 5]. These epigenetic defects are linked with genomic instability, MM progression, high-risk disease and more recently also the development of DR [3, 6]. For instance, a clear role has been established for the histone methyltransferases (HMTs) multiple myeloma SET domain (MMSET) and enhancer of zeste 2 (EZH2) in the DR against the alkylating agent melphalan and PIs/IMiDs respectively [7, 8]. In addition, resistance against the anti-CD38 moAbs daratumumab (Dara) and isatuximab (Isa) has recently been linked with epigenetic silencing of *CD38* through EZH2, HDAC6 and/or KDM6A loss. Accordingly, histone deacetylase inhibitors (HDACi, panobinostat), DNA methyltransferase inhibitors (DNMTi, decitabine (DAC) and azacytidine) and EZH2 inhibitors were shown to restore sensitivity to these moAbs [9–12]. Finally, acquired resistance to GPRC5D-directed T-cell engagers has also been linked with antigen escape due to epigenetic silencing [13]. This underscores the potential of epigenetic reprogramming of the MM cells using epigenetic modulating agents (EMAs) to (re)sensitize them not only to the current standard of care (SOC) agents, but also to emerging immunotherapies. However, the high toxicity profiles of the broad-acting EMAs (the HDACi and DNMTi) together with the knowledge-gap about which epiplayers are key in driving MM relapse remain two major limiting factors [3, 14].

With the aim of identifying new, clinically relevant epiplayers driving relapse in MM, we recently consulted the publicly available RNA-Seq data from matched newly diagnosed and relapsed MM patients from the MMRF CoMMpass study. We observed that DNA methyltransferase 3B (*DNMT3B*) expression is significantly increased in relapsed patients compared to newly diagnosed patients. DNMT3B is a member of the DNMT family that consists of five members, including DNMT1, DNMT2, DNMT3A, DNMT3B and DNMT3L, among which only DNMT1, DNMT3A and DNMT3B are catalytically active [15]. DNMT1 is mainly involved in maintenance of already existing DNA methylation patterns in differentiated cells, while DNMT3A and DNMT3B are highly expressed in undifferentiated embryonic stem cells and are the ones establishing new methylation patterns [5, 16]. *DNMT3B* overexpression due to miR-29a/b downregulation and/or MYC overexpression is observed in several solid and hematological cancers and is linked with poor prognosis [17]. Moreover, DNMT3B targeting using genetic depletion or miR-29 mimics reduces cancer cell growth and survival in several tumor models, thereby confirming its oncogenic role [5, 17, 18]. However, some studies have also reported the opposite. In acute myeloid leukemia (AML) for example, *Dnmt3b* deletion led to accelerated progression in an MLL-AF9 driven mouse model [19]. Hence, the role of DNMT3B appears cell-context dependent. In MM, increased *DNMT3A/B* levels in MM patients compared to patients with the premalignant condition monoclonal gammopathy of undetermined significance (MGUS) due to miR29a/b downregulation have been reported and miR-29b mimics were shown to inhibit MM cell growth and promote cell killing by bortezomib and CD8 + T cells [20, 21]. Furthermore, a recent study showed that granulocytic myeloid-derived suppressor cells increase piRNA-823 and DNMT3B levels in MM cells, which was suggested to result in an enhanced MM stemness potential [22]. Together, these studies point to an oncogenic role for DNMT3B in MM. Nonetheless, the exact role of DNMT3B in MM biology and drug response remains poorly defined.

## Materials and methods

### Gene expression profiling data

Expression and survival analysis of gene expression profiling (GEP) data was performed using Genomicscape (http://genomicscape.com*)* and Graphpad Prism 8 software. DNMT3B mRNA levels between newly diagnosed and relapsed samples were compared using the publicly available RNA-Seq data of the CoMMpass study (https://research.themmrf.org/, release IA12, NCT01454297). The prognostic value of DNMT3B was evaluated in newly diagnosed patients from the CoMMpass study and UAMS TT3 cohort (GSE2658) and relapsed patients subsequently treated with bortezomib (Mulligan cohort; GSE9782) or daratumumab (Dara cohort) [11, 23–28]. We also compared DNMT3B levels in BMPC, MM and HMCL using our previously generated RNA-seq data [23, 28, 29].

### Compounds

Nanaomycin A (NA; 10 mM) was purchased from Gentaur (Kampenhout, Belgium), while bortezomib (Bz; 10 mM), melphalan (Mel; 5 mM), decitabine (DAC; 10 mM) and puromycin (50 mg/mL) were obtained from Selleckchem (Munich, Germany). Blasticidin S HCl (Bsd; 10 mg/mL) and MG132 (10 nM) were obtained from Gibco - Thermofisher Scientific. Doxycycline (10 mg/mL) was obtained from Sigma-Aldrich (Saint Louis, Missouri, USA), while Dimethyl Sulfoxide (DMSO) was obtained from MP-Biomedicals (Santa Ana, California, USA) and used as solvent for all compounds mentioned above except for doxycycline, which was dissolved in 1x PBS. Stock solutions were stored at -20 °C. Daratumumab (Dara; 5.09 mg/mL) and Isatuximab (Isa; 6.5 mg/mL) were purchased from Selleckchem and stored in the dark at 4 °C. Cycloheximide (CHX; 10 mg/mL) was purchased from Cell Signaling Technology (Leiden, The Netherlands) and dissolved in 1x PBS and stored in the dark at -20 °C.

### DNMT3B genetic depletion

AMO-1 and XG-2 cells were transduced (MOI of 10) with 3 different SMARTvector inducible Lentiviral shRNA vectors (Horizon Discovery, Waterbeach, United Kingdom), containing a doxycycline inducible shRNA against human *DNMT3B* (*shDNMT3B*). Clone IDs are listed in Suppl. Table 1. Transduced cells were selected using 4 µg/mL puromycin and further subcloned to obtain uniform clones with strong *DNMT3B* knockdown. Expression of *shDNMT3B* and eGFP was achieved by adding 1 µg/mL doxycycline.

### MYC overexpression

The sh cont and sh1.2 XG-2 cells and U266 cells were transduced (MOI of 10) with a lentiviral vector (VectorBuilder, Neu-Isenburg, Germany) containing the human c-MYC gene (c-*MYC*) under the control of an EF1A promotor. Transduced cells were selected using 2 µg/mL Bsd.

### RNA sequencing

Total RNA was extracted and purified using the NucleoSpin RNA plus kit (Macherey-Nagel, Düren, Germany) and RNA concentrations were measured using the Nanodrop-1000 (Thermofisher). Sequencing libraries were prepared using the Illumina TruSeq Stranded mRNA Library Prep Kit and 150 ng RNA. The Illumina NextSeq system (Helixio, Clermont-Ferrand, France) and STAR aligner were used for paired-end sequencing analysis and alignment of the RNA-Seq reads to the reference human GRCh37 genome respectively. Software R (version 4.2.2) and its R packages were used to perform statistical analyses [30]. The DESeq2 R/Bioconductor package (version 1.38.3) was used to summarize and normalize the expression level of each gene [31]. P-values were adjusted to control global FDR across all comparisons with the default option. All genes with an adjusted P-value of 0.05 and a fold change of 1.5 were considered differentially expressed.

### Statistical analysis

Statistical analysis was performed using GraphPad Prism 8.0 software and Genomicscape. Differences between two groups were evaluated using either a one-tailed Mann-Whitney U test or a Wilcoxon matched-pairs signed rank test for paired analysis, while a one-way ANOVA test was used to compare more than two groups. Multivariate analysis was performed using multivariate cox regression analysis. P-values of ≤ 0.05 were considered statistically significant. For the GEP data, all raw CEL files were gcrma-normalized in the same manner in R using Bioconductor (gcrma package). Gene set enrichment analysis (GSEA) was performed in R (dplyr, fgsea and ggplot2 packages) using the hallmark and the curated gene sets (C2) from the Molecular Signatures Database.

A detailed description of all other materials and methods is available in ‘Supplementary Material 1’.

## Results

### DNMT3B levels are increased in the relapsed setting and high DNMT3B levels are associated with aggressive disease and a worse outcome

**Fig. 1:**
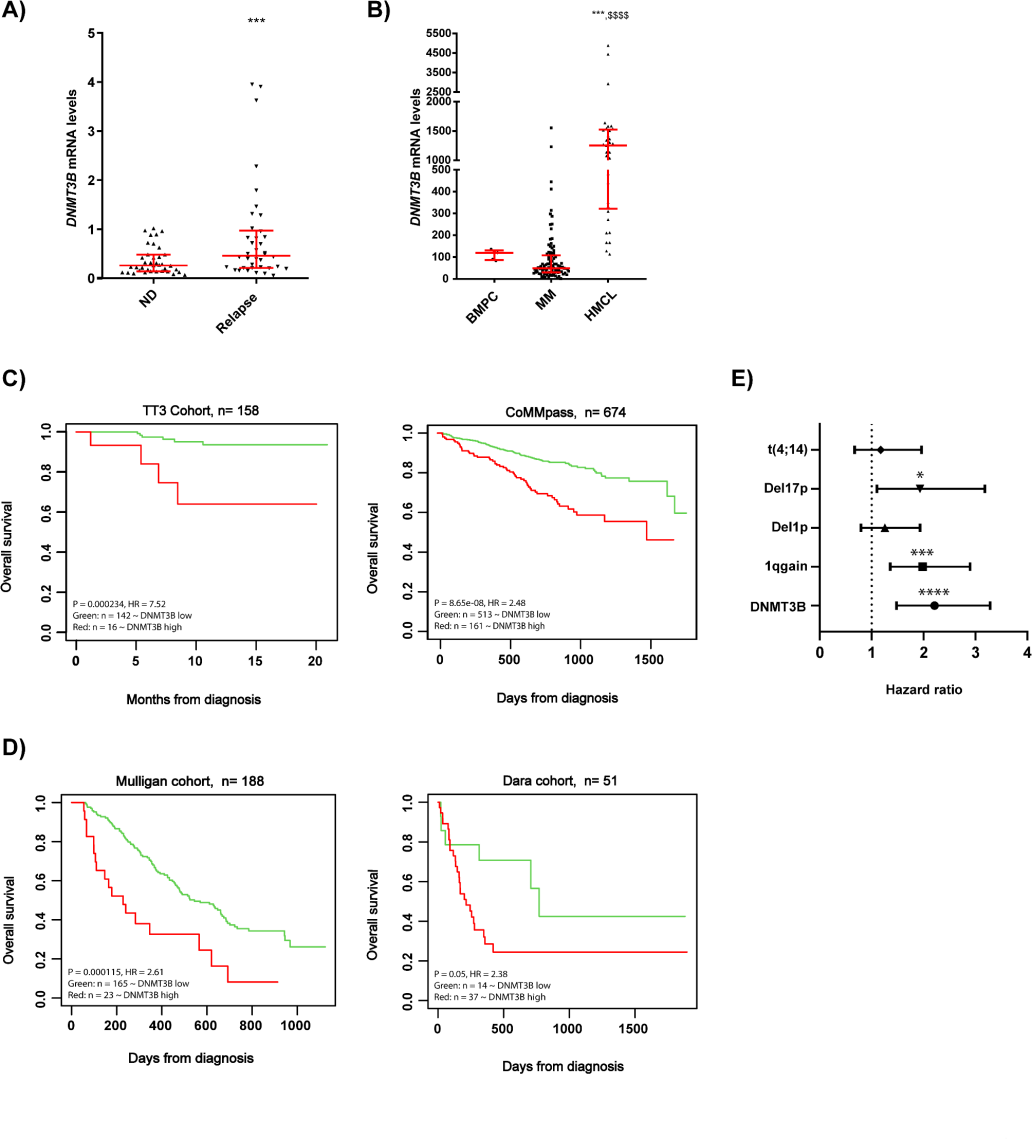
Expression and prognostic value of *DNMT3B* in MM. (**A**) Comparison of the *DNMT3B* mRNA levels of matched newly diagnosed (ND) and relapsed (Relapse) primary samples (*n* = 38) from the CoMMpass study. ***p ≤ 0.001. (**B**) *DNMT3B* mRNA levels as determined by RNA-Seq in normal bone marrow plasma cells (PCs, *n* = 5), primary MM cells (*n* = 97) and HMCLs (*n* = 33). ***p ≤ 0.001 compared to PCs, *p* ≤ 0.0001 compared to MM cells. **C**-**D**) Prognostic value of *DNMT3B* mRNA in terms of overall survival (OS) in ND (TT3 and CoMMpass cohort, **C**) and relapsed patients (Mulligan and Dara cohort, **D**). Maxstat analysis was used to calculate the optimal separation of patients based on a cut-off value. **E)** Multivariate cox analysis of DNMT3B, del17p, 1q gain, del 1p and t(4;14) using the data from the CoMMpass study. This forest plot shows the hazard ratios (HR) ± 95% CI. *p ≤ 0.005, ***p ≤ 0.001 and ****p ≤ 0.0001

Using the RNA-Seq data from matched newly diagnosed (ND) and relapsed patient samples from the CoMMpass study, we found that *DNMT3B* is significantly upregulated in relapsed samples (*p* < 0.001; Fig. 1A). Moreover, we found a significant increase in *DNMT3B* levels in human myeloma cell lines (HMCLs) compared to primary MM cells (*p* < 0.001) and normal plasma cells (PCs; *p* < 0.0001; Fig. 1B; Figure S1A). Looking in more detail into the MM group, we noticed a quite heterogeneous *DNMT3B* expression, with some patients having fairly high levels compared to normal PCs. Zooming further in into the different molecular subsets, *DNMT3B* levels were significantly elevated in the proliferation (PR; *p* ≤ 0.001), cyclin D1 (CD-1; *p* ≤ 0.005) and cyclin D3 (CD-2; *p* < 0.005) overexpressing group and significantly lower in the low bone disease (LB; *p* ≤ 0.05) and hyperdiploid (HY; *p* ≤ 0.005) group (Figure S1B) [32]. Importantly, *DNMT3B* was also high in patients categorized in the gene expression-based proliferation index 3 (GPI3) group (*p* ≤ 0.0001) and patients harbouring a TP53 mutation or del17 (*p* ≤ 0.05), all of which are associated with high proliferation and/or poor prognosis (Figure S1C-E) [33–35]. When evaluating the prognostic value of DNMT3B in both ND and relapsed patients, we observed a significantly worse outcome in patients with high *DNMT3B* levels compared to patients with low *DNMT3B* levels, with significant lower progression free and overall survival rates (Fig. 1C-D; Figure S1F-G) [11]. Finally, when testing the prognostic value of DNMT3B, del17p, 1q gain, del1p and t(4;14) all together in the CoMMpass study, we found that DNMT3B (*p* ≤ 0.0001), del17p (*p* ≤ 0.05) and 1q gain (*p* ≤ 0.001) remained independent prognostic factors (Fig. 1E).

### DNMT3B is heterogeneously expressed in human MM cell lines and patient samples

**Fig. 2:**
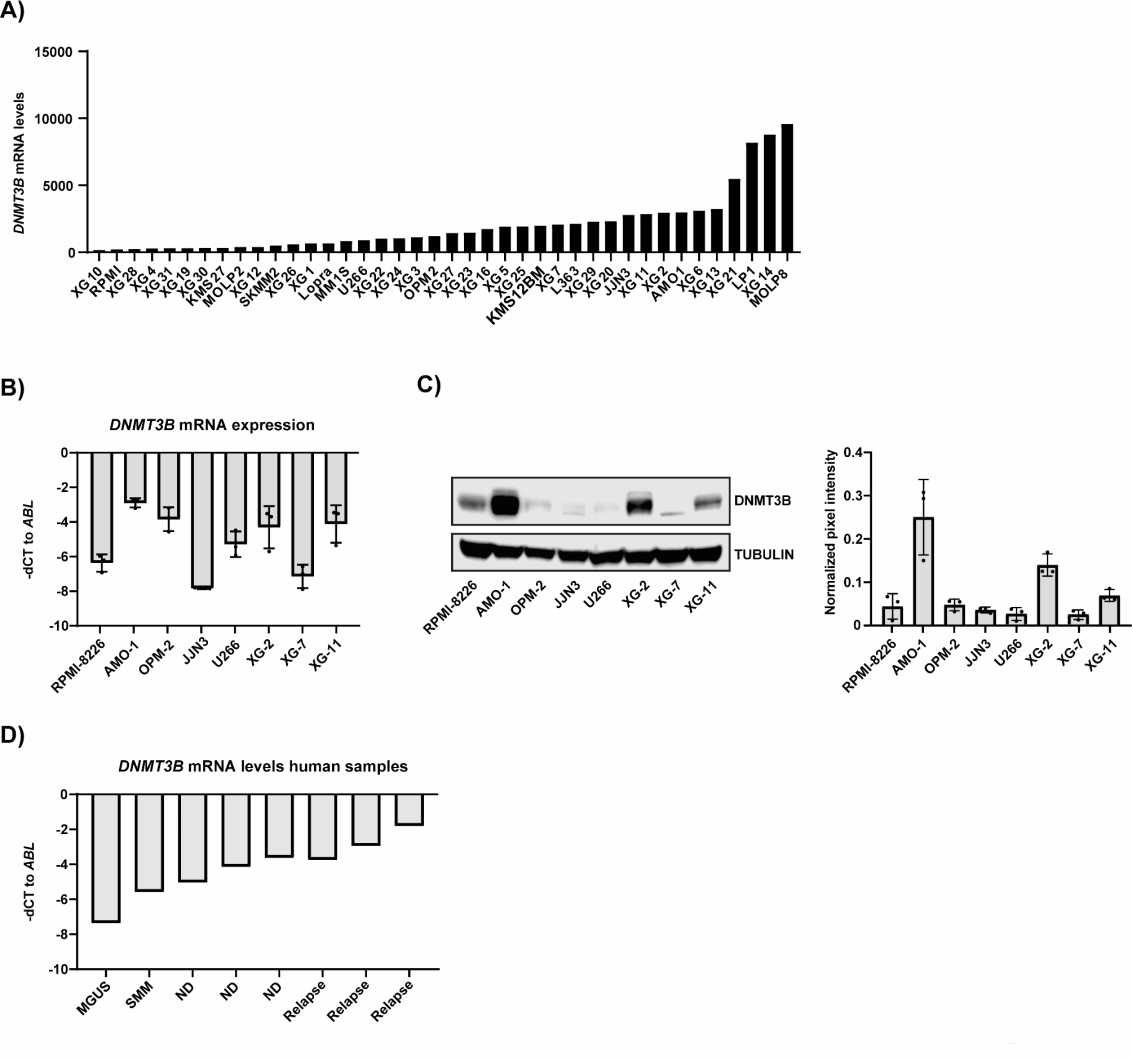
*DNMT3B* expression in human MM cell lines and patient samples. **A**) *DNMT3B* mRNA levels in 40 different HMCLs using our own RNA-Seq data. **B**) *DNMT3B* mRNA expression in a selected panel of HMCLs using qRT-PCR. *ABL* was used as a reference gene. The mean expression ± SD for three independent experiments is shown. **C**) DNMT3B protein expression determined in a selected panel of HMCLs via western blot. Tubulin was used as loading control. Left: blots of one experiment representative of three are shown, right: quantification of the DNMT3B levels relative to tubulin as measured by Image Studio for the 3 independent experiments. **D**) The *DNMT3B* levels in PCs obtained from one monoclonal gammopathy of undetermined significance (MGUS), one smoldering myeloma (SMM), 3 newly diagnosed (ND) and 3 relapsed (Relapse) MM patients were determined using qRT-PCR. *ABL* was used as reference gene.

Next, we analysed *DNMT1*, *DNMT3A* and *DNMT3B* mRNA levels in 40 HMCLs using our previously generated RNA-Seq data [23]. In general, we observed high basal *DNMT1* levels, intermediate *DNMT3B* levels and low levels of *DNMT3A* (Fig. 2A; Figure S2A, D). Moreover, the mRNA levels were also quite heterogeneous for *DNMT3A/B* among the different HMCLs. This heterogeneous expression was validated on mRNA and protein level for a selected panel of HMCLs. For DNMT3B, we observed high levels in AMO-1, intermediate levels in XG-2 and XG-11 and low levels in RPMI-8226, OPM-2, JJN3, U266 and XG-7 (Fig. 2B-C). In contrast, DNMT1 levels were quite similar, whereas DNTM3A was undetectable on protein level (Figure S2B-C, E). Finally, we also evaluated DNMT3B mRNA levels in primary samples of monoclonal gammopathy of undetermined significance (MGUS; *n* = 1), smoldering myeloma (SMM; *n* = 1), ND (*n* = 3) and relapsed (*n* = 3) MM patients using qPCR. In line with the GEP data, we observed a clear increase in DNMT3B levels upon disease progression (Fig. 2D).

### Genetic depletion of DNMT3B impairs MM cell growth, survival and clonogenicity

**Fig. 3:**
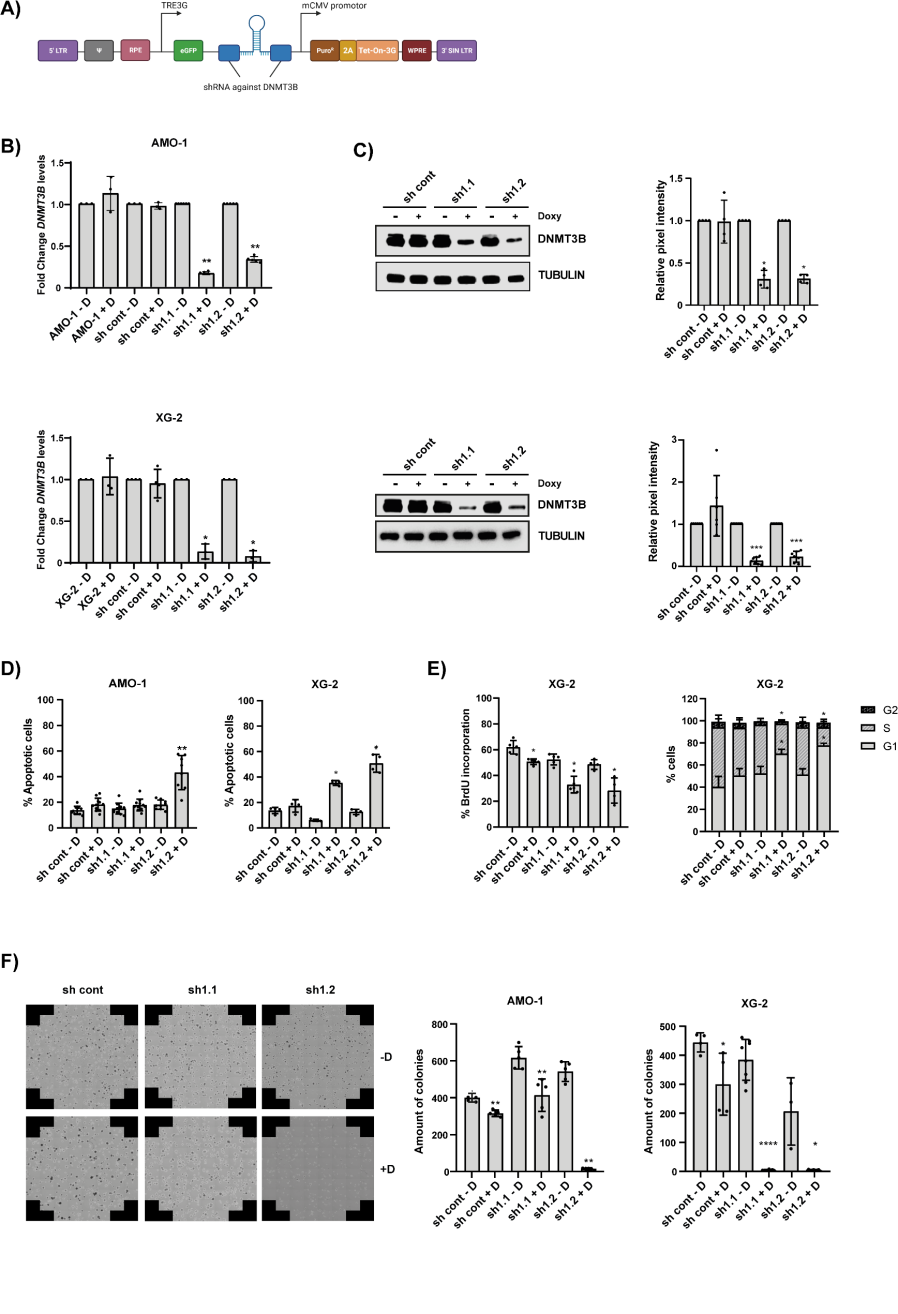
Effect of *DNMT3B* knockdown on MM cell biology. (**A**) Visual representation of the inducible lentiviral vectors containing a shRNA cassette against *DNMT3B* (*shDNMT3B*). (**B**) Validation of *DNMT3B* knockdown (KD) in the AMO-1 (upper panel) and XG-2 (lower panel) cells on mRNA level. *DNMT3B* levels were determined after 3 days of doxycycline treatment using qPCR. *ABL* was used as reference gene. The relative expression levels in stimulated (+ D) compared to unstimulated (-D) cells are shown (*n* = 3). (**C**) Validation of DNMT3B KD in AMO-1 (upper panel) and XG-2 (lower panel) cells on protein level. DNMT3B levels were determined by western blot 5 days post-doxycycline treatment. Tubulin was used as loading control. Left: one experiment representative of at least three is shown, right: quantification of DNMT3B levels relative to tubulin as measured by Image Studio and normalized to unstimulated (-D) cells. **D**-**F**) Effect of *DNMT3B* KD on apoptosis (**D**), proliferation (**E**) and clonogenic outgrowth (**F**). (**D**) Cells were stimulated for 5 days with or without doxycycline and apoptosis was measured by an AnnexinV/7’AAD staining followed by flow cytometric analysis. The % apoptotic cells are the sum of AnnexinV (+) and AnnexinV (+)/7’AAD (+) cells. (**E**) Cells were treated for 3 days with doxycycline after which the effect on bromo-deoxyuridine (BrdU) incorporation (left panel) and cell cycle progression (right panel) was determined using BrdU and PI-stainings respectively. (**F**) Transduced AMO-1 and XG-2 cells were treated for 5 or 3 days respectively with doxycyline and were then plated to perform a colony forming assay. The number of colonies were determined after 14 days using the EVOS M7000 Imaging System (left) and counted with ImageJ software (right). The mean ± SD of at least three independent experiments is shown. *p ≤ 0.05, **p < 0.01, ***p < 0.001 and ****p < 0.0001 compared to unstimulated (-D) cells

We next evaluated the functional role of DNMT3B in MM, by knocking down DNMT3B (DNMT3B KD) in AMO-1 and XG-2 cells using inducible lentiviral vectors containing shRNAs against *DNMT3B* (sh1-3; Fig. 3A; Table S1). All three *shDNMT3Bs* reduced DNMT3B mRNA and protein levels upon doxycycline treatment while leaving DNMT1 unaffected, with the strongest reduction obtained for sh1 (Figure S3A-D). To obtain uniform clones of transduced cells, we subsequently performed subcloning for the HMCL transduced with sh1. Two subclones per cell line with at least 70% KD on protein level, namely sh1.1 and sh1.2, were selected for subsequent experiments (Fig. 3B-C; Figure S3E-F). As illustrated in Fig. 3D, we observed a strong increase in the percentage of apoptotic cells upon *DNMT3B* KD. Moreover, *DNMT3B* genetic depletion resulted in reduced BrdU uptake and an arrest in the G1-phase (Fig. 3D-E). Finally, we evaluated if *DNMT3B* KD is also able to affect the ability of seeded single cells to produce colonies (hence undergo ‘unlimited’ division). As shown in Fig. 3F, we observed a strong and significant reduction in the number of colonies upon *DNMT3B KD*.

### DNMT3B depletion mainly affects programs involved in cell cycle regulation and stemness

**Fig. 4:**
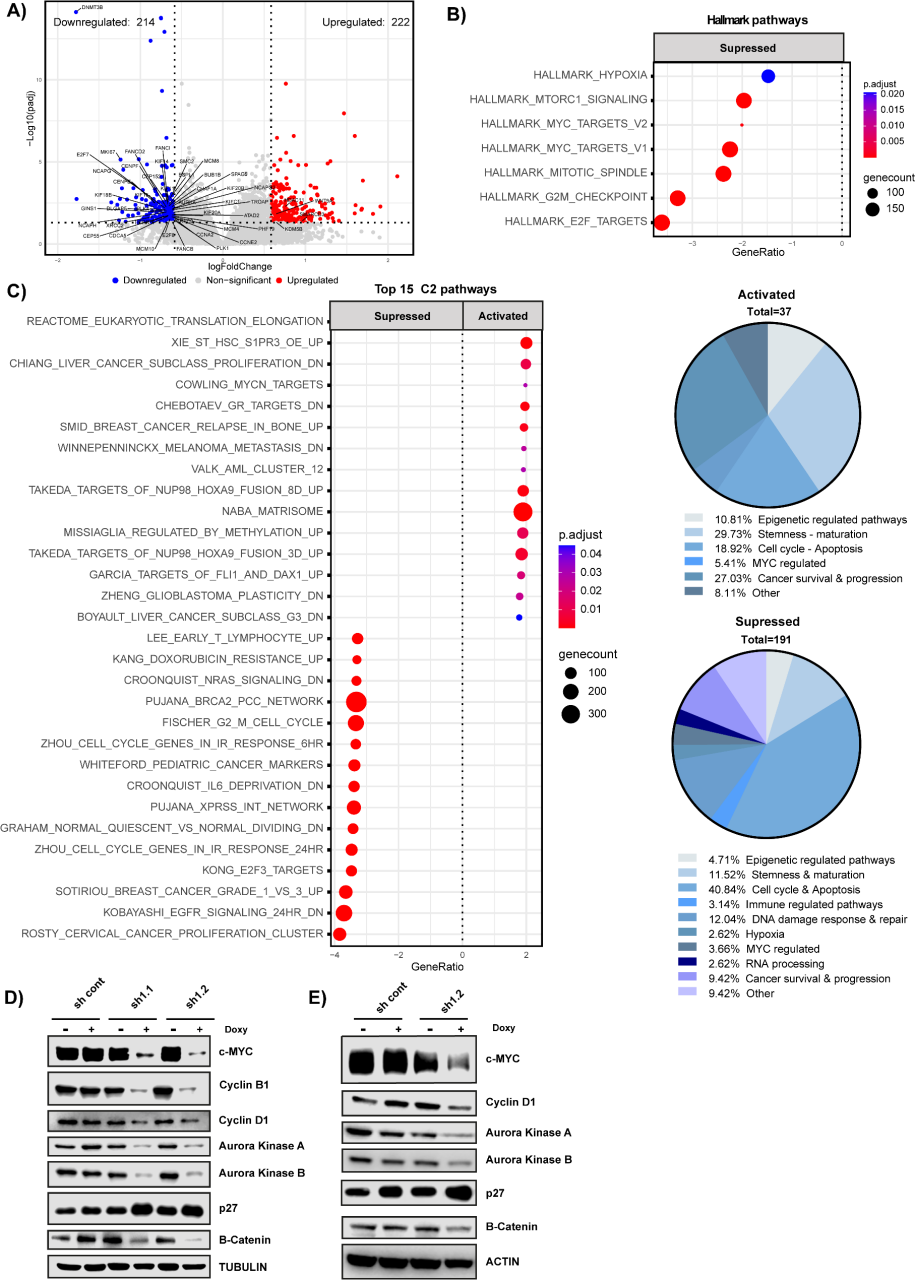
Transcriptomic analysis upon DNMT3B depletion. (**A**) Volcano plot showing upregulated (red) and downregulated (blue) genes upon DNMT3B KD. (**B**) Molecular signatures of suppressed genes upon *DNMT3B* KD after GSEA using the hallmark sets. (**C**) Left panel: Top 15 molecular signatures of the suppressed and activated genes upon DNMT3B KD after GSEA using all curated sets (C2). Right panel: pie charts showing the percentage of pathways involved in cell cycle, apoptosis, stemness, epigenetic regulated pathways or other. **D**-**E**) Protein levels of indicated cell cycle regulators determined by western blot for the *shDNMT3B* XG-2 (**D**) and *shDNMT3B* AMO-1 (**E**) cell line. Tubulin or actin was used as loading control. One experiment representative of three is shown. Quantification of the WB data is provided in Figure S4A-B

To reveal transcriptional programs affected by *DNMT3B* KD in MM cells, we next performed transcriptomic analysis. We identified 436 genes differentially expressed in AMO-1 and XG-2 *DNMT3B*-depleted cells (Fold change > 1.5; FDR ≤ 0.05); with 222 genes upregulated and 214 genes downregulated (Fig. 4A; Table S2). GSEA using the Hallmark gene sets showed that genes involved in mitotic spindle, MYC targets, MTORC1 signaling and cell cycle related pathways were significantly less enriched in DNMT3B-depleted cells (Fig. 4B; Table S3). GSEA using the curated gene sets (C2) confirmed this, showing that upregulated genes are mainly involved in epigenetic regulated pathways (10.81%), stemness - maturation (29.73%), cell cycle - apoptosis (18.92%) and cancer survival and progression (27.03%), whereas suppressed genes are involved in epigenetic regulated pathways (4.72%), stemness - maturation (11.52%), cell cycle - apoptosis (40.84%), immune regulated pathways (3.14%), DNA damage response - repair (12.04%), hypoxia (2.62%), MYC regulated pathways (3.66%), RNA processing (2.62%) and cancer survival - progression (9.42%) (Fig. 4C; Table S4). We validated these findings on protein level using WB. As illustrated in Fig. 4D-E and Figure S4, the cell cycle activators c-MYC, Cyclin B1, Cyclin D1, Aurora kinase A and B were all significantly and consistently downregulated upon DNMT3B KD, whereas the cell cycle inhibitor p27 was upregulated. Furthermore, we also observed a significant decrease in β-catenin levels, linked with stemness.

### DNMT3B depletion is also effective in killing MM cells with high basal c-MYC levels

**Fig. 5:**
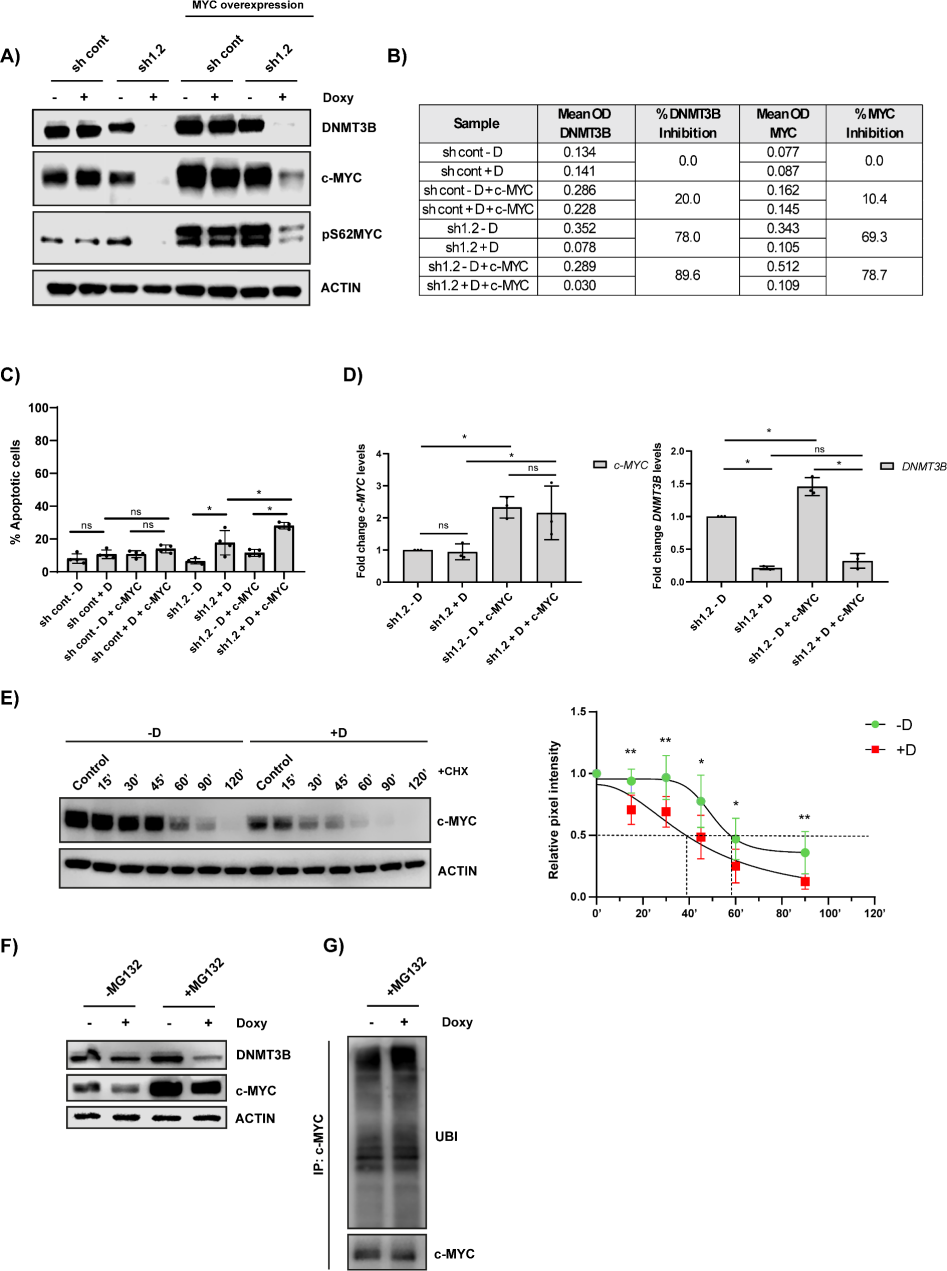
Effect of c-MYC overexpression on *DNMT3B* depletion outcome. (**A**) DNMT3B, c-MYC and pS62MYC protein levels upon *DNMT3B* depletion in XG-2 cells with or without constitutive c-MYC overexpression were determined after 3 days of doxycycline treatment using western blot. Actin was used as loading control. One experiment representative of at least three is shown. (**B**) Mean OD values and % inhibition of c-MYC and DNMT3B levels. (**C**) Cells were stimulated for 3 days with doxycycline and apoptosis was measured by an AnnexinV/7’AAD staining followed by flow cytometric analysis. The % apoptotic cells are the sum of AnnexinV (+) and AnnexinV (+)/7’AAD (+) cells. Bars are the mean ± SD of at least three independent experiments. *p ≤ 0.05. (**D**) c-*MYC* and *DNMT3B* levels in *shDNMT3B* transduced XG-2 cells with and without c-MYC overexpression after 3 days of doxycycline treatment using qRT-PCR. *ABL* was used as reference gene. (**E**) c-MYC protein stability upon *DNMT3B* depletion. XG-2 sh1.2 cells were treated with or without doxycycline for 24 h after which cycloheximide (CHX; 50 µg/mL) was added for indicated timepoints. Left: one experiment representative of at least four is shown, right: pixel density of the bands obtained for c-MYC relative to actin as measured by Image Studio and normalized to control. The mean ± SD of at least four independent experiments is shown. *p ≤ 0.05 and **p ≤ 0.01 compared to -D. (**F**) DNMT3B and c-MYC protein levels of XG-2 sh1.2 cells treated with doxycyline for 24 h followed by MG132 treatment (5 µM) for an additional 3 h. (**G**) Ubiquitinated levels of c-MYC of XG-2 sh1.2 cells treated with doxycyline for 24 h followed by MG132 (5 µM) treatment for an additional 3 h

Since DNMT3B depletion strongly reduced c-MYC levels, a key oncogene overexpressed in about 70% of MM patients and linked with progression and adverse outcomes [36–38], and affected the expression levels of MYC targets, we next evaluated if DNMT3B targeting could be of benefit to patients with deregulated MYC. Therefore, we next evaluated the effect of constitutive c-MYC overexpression on *DNMT3B* depletion outcome. As shown in Fig. 5A-B and Fig. 5SA, c-MYC overexpression did not affect the level of *DNMT3B* silencing nor did it affect the potential of *DNMT3B* KD to decrease c-MYC levels, with the % downregulation of DNMT3B and c-MYC levels being similar, or even slightly higher, than in non-c-MYC overexpressing cells. Moreover, *DNMT3B* depletion was also slightly more potent in inducing apoptosis after 3 days of doxycyline treatment in c-MYC overexpressing compared to non-c-MYC overexpressing cells (Fig. 5C). Surprisingly, *DNMT3B* depletion had only minor effects on c-*MYC* mRNA levels, indicating that DNMT3B is supporting c-MYC’s stability rather than regulating its transcription (Fig. 5D). Indeed, using the cycloheximide (CHX) chase assay, we found that c-MYC levels declined more rapidly in DNMT3B-depleted cells after 24 h of doxycycline stimulation (t1/2 of 38 min in + D compared to 58 min for -D), a timepoint where we had stable eGFP expression and *DNMT3B* silencing but not yet cell death (Fig. 5E, Figure S5B-D). Notably, we also observed a significant decrease in phosphorylation of c-MYC at serine 62 (pS62MYC, Fig. 5A), which is well known to prime MYC for proteasomal degradation [39–41], suggesting that DNMT3B protects c-MYC from proteasomal degradation. In accordance, ubiquitinated c-MYC levels were found elevated in DNMT3B-depleted cells following treatment with the PI MG132, while increased c-MYC turnover was partially rescued (Fig. 5F-G and Figure S5E).

### Nanaomycin A supports the therapeutic potential of DNMT3B targeting in MM

**Fig. 6:**
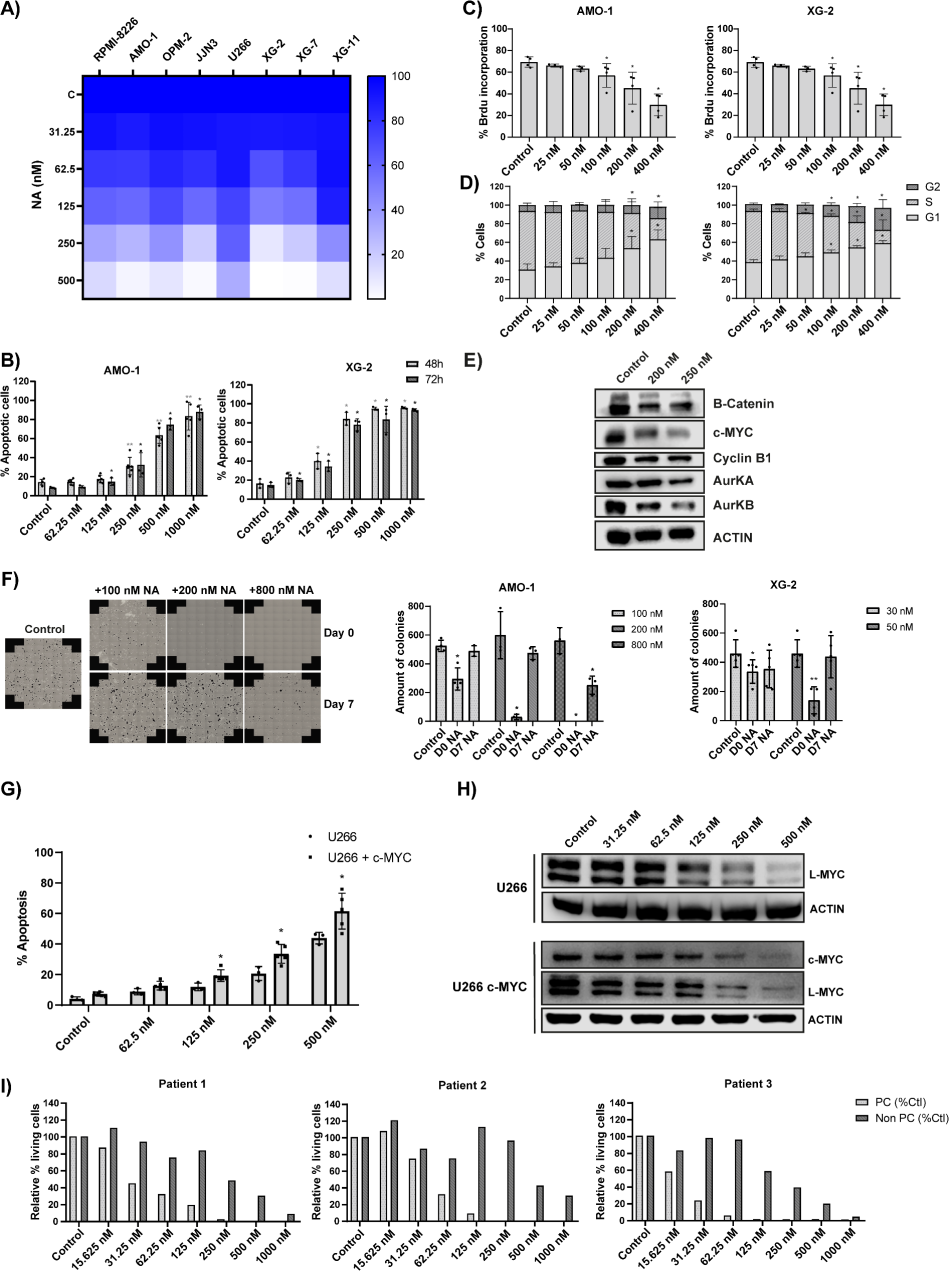
Effect of Nanaomycin A on human MM cell lines and patient cells. (**A**) Effect of NA on HMCL viability after 72 h. (**B**) Apoptosis in AMO-1 and XG-2 evaluated after 48 h (light gray) and 72 h (dark gray). **C**-**D**) Effect of NA treatment on BrdU incorporation (**C**) and cell cycle progression (**D**) after 24 h. **E**) Protein levels of indicated cell cycle regulators in the XG-2 cells following 3 days of NA treatment (200 and 250 nM). Actin was used as loading control. One experiment representative of three is shown. **F**) Effect of NA on MM cell clonogenicity. AMO-1 cells were treated with 100, 200 nM (low doses) or 800 nM (high dose) of NA on the day of plating (day 0) or 7 days post-plating (day 7), whereas XG-2 cells were treated with 30 and 50 nM (low doses) of NA either on day 0 or day 7. Colonies were counted 14 days post-plating using the EVOS M7000 Imaging System. Left: visual representation of the AMO-1 colony forming assay, right: number of colonies counted with ImageJ. The mean ± SD of at least three independent experiments is shown. *p ≤ 0.05 and **p ≤ 0.01 compared to control. **G**) Effect of NA on apoptosis in the wildtype and c-MYC overexpressing U266 cells after 72 h of treatment. **H**) Protein levels of c/L-MYC upon 3 days of NA treatment of the wildtype (upper) and c-MYC U266 overexpressing (lower) cells. Actin was used as loading control. One experiment representative of three is shown. **I**) Effect of NA on primary human samples. Purified primary BM mononuclear cells (*n* = 3) were treated for 4 days with indicated concentrations of NA and the percentage of viable malignant CD138 + plasma cells (PC) and non-tumoral CD138- cells (Non PC) was evaluated by flow cytometric analysis

To validate the potential of DNMT3B as a therapeutic target in MM, we next evaluated the anti-MM activity of the DNMT3B selective small molecule inhibitor Nanaomycin A (NA). NA is a quinone antibiotics that selectively targets DNMT3B by directly interacting with its catalytic site, thereby reducing global DNA methylation and inducing cytotoxicity in several cancer cell lines [42]. In line with the loss-of-function studies, we observed a significant and dose-dependent decrease in viability for all cell lines tested, with the cell lines with the highest basal DNMT3B levels, AMO-1 and XG-2, showing the highest sensitivity. Moreover, we observed a significant and dose-dependent increase in apoptosis of AMO-1, XG-2 and XG-7 cells upon NA treatment (Fig. 6A-B and Figure S6A). Low doses of NA also impaired MM cell proliferation, as evidenced by a G1/G2 phase arrest, reduced BrdU uptake and reduced levels of the cell cycle activators (including c-MYC), as well as their clonogenic capacity (Fig. 6C-F, Figure S6B-C). Importantly, for the latter, we observed a significant and dose-dependent decrease in the number of colonies when low doses of NA were added on the day of plating, but not when added 7 days after plating (Fig. 6F). The reduced number of colonies was not due to cytotoxic activity, as treatment with low doses of NA (range 12.5 − 100 nM) for up to 14 days did not result in clear apoptosis (Figure S7A). In contrast, treatment with a high dose significantly reduced the number of colonies when added at both timepoints (Fig. 6F). Comparable effects were observed after treatment with the pan-DNMTi DAC (Figure S7B). Importantly, in line with DNMT3B KD, we found that AMO-1 and XG-2 c-MYC-overexpressing cells were more sensitive to NA treatment compared to their normal counterparts, suggesting that an active c-MYC transcriptional program sensitizes MM cells to DNMT3B targeting (Figure S8A). To address this further, we next overexpressed c-MYC in U266 cells that are defective for c-MYC (c-MYC null cells) but express high levels of L-MYC instead (Figure S8B) [43–46], and evaluated the effect of NA on apoptosis and c/L-MYC protein levels. NA also induced apoptosis in U266 wildtype cells, albeit to a lesser extent compared to the other cell lines, while dose-dependently reducing L-MYC levels (Fig. 6G-H and Figure S8C). More importantly, NA was again considerably more cytotoxic in c-MYC overexpressing cells than wildtype cells, reducing both c-MYC and L-MYC protein levels (Fig. 6G-H and Figure S6C-D). Finally, we also validated the anti-MM activity of NA on primary patient samples (*n* = 9; Table S5). As shown in Fig. 6I and Fig. S9A-B, primary CD138 + MM cells (PC) were found highly sensitive to NA, both when purified or co-cultured with the non-tumoral (CD138-) bone marrow (BM) stromal cells (non-PC). In contrast, both the non-PC fraction and cultured primary human BM stromal cells were much less affected. Notably, primary samples with high DNMT3B levels, defined by a large proportion of CD138 + cells with high DNMT3B mRNA copy numbers, showed increased sensitivity to NA treatment compared to DNMT3B low samples (Figure S9C-D).

### Nanaomycin A (re)sensitizes MM cells to bortezomib, melphalan and anti-CD38 monoclonal antibodies

**Fig. 7:**
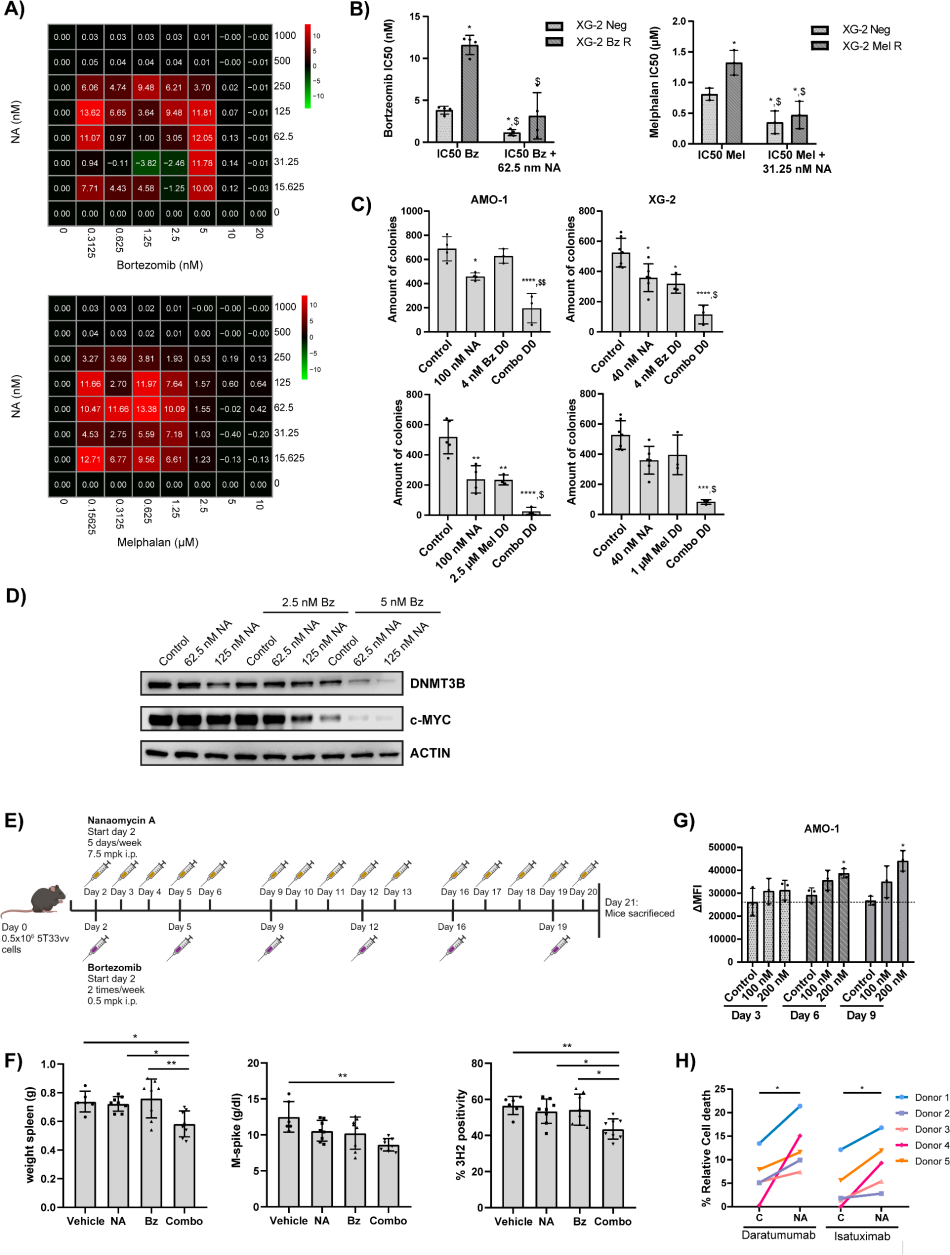
Nanaomycin A sensitizes MM cells to bortezomib, melphalan and anti-CD38 monoclonal antibodies. (**A**) Effect of NA on bortezomib (Bz) or melphalan (Mel) sensitivity. XG-2 cells were treated 4 days with NA and/or Bz or Mel and the effect on viability was assessed. Synergy scores were calculated using the Bliss method. The mean of at least three independent experiments is shown. (**B**) Effect of NA on Bz or Mel sensitivity in resistant cell lines. XG-2 parental (XG-2 Neg) and Bz/Mel resistant (XG-2 Bz R/Mel R) cells were treated 4 days with the IC50 of the parental cells respective standard of care agent alone or in combination with 62.5 nM or 31.25 nM NA respectively. The mean ± SD of at least three independent experiments is shown. *p ≤ 0.05 compared to IC50 Bz/Mel XG-2 Neg. $ *p* ≤ 0.05 compared to IC50 Bz/mel XG-2 Bz/Mel R. (**C**) Effect of combination treatment on clonogenic outgrowth. AMO-1 and XG-2 cells were treated with NA and/or Bz/Mel on day 0. Colonies were counted 14 days post-plating using the EVOS M7000 and ImageJ. Mean ± SD of at least three independent experiments is shown. *p ≤ 0.05, **p ≤ 0.01, ***p ≤ 0.001 and ****p < 0.0001 compared to control. $ *p* ≤ 0.05 and $$ *p* ≤ 0.01 compared to both single agents alone. (**D**) DNMT3B and c-MYC protein levels in XG-2 cells after 24 h of NA and/or Bz treatment. Actin was used as loading control. One experiment representative of three is shown. (**E**) Set-up of the 5T33MM mouse experiment. (**F**) Effect of low dose NA and/or Bz treatment on spleen weight, M-protein serum levels and the percentage of 3H2-positive myeloma cells. *p ≤ 0.05, **p ≤ 0.01. **G**-**H** Effect of long-term low dose NA treatment (up to 9 days) on CD38 cell surface expression (**G**) and ADCC induced by daratumumab (1 µg/mL) and isatuximab (10 ng/mL) (**H**) for the AMO-1 cells. NK cells were added with an effector-to-target ratio of 5:1. *p ≤ 0.05 compared to control

We finally evaluated whether DNMT3B influences MM cell responses towards SoC agents. Combining NA with either bortezomib (Bz) or melphalan (Mel) indeed resulted in synergistic anti-myeloma activity (Fig. 7A; Figure S10A). Moreover, NA was also able to resensitize Bz and Mel resistant XG-2 cells to Bz and Mel respectively (Fig. 7B). Combination of a low dose of NA with a low dose of Bz or Mel furthermore resulted in a significant decrease in colony outgrowth compared to NA or Bz/Mel treatment alone (Fig. 7C), supporting the notion that DNMT3B supports clonogenic outgrowth of residual cells upon Bz or Mel treatment. For Bz, combination treatment was found to further reduce both DNMT3B and c-MYC levels compared to the single agents (Fig. 7D). Moreover, the enhanced anti-myeloma effects were also validated in XG-2 DNMT3B KD cells (Figure S10B) and in the murine 5T33MM model (Fig. 7E), for which the primary tumor cells (5T33MMvv) were again found much more sensitive to NA treatment than the BMSC (Figure S11A-C). In line with the HMCL data, 5T33MM mice treated with a suboptimal dose of NA and Bz showed a significant decrease in spleen weight, M-spike and % of 3H2-positive MM cells as compared to single agent treated mice (Fig. 7F). None of the mice showed overt signs of toxicity, as evidenced by having a normal behaviour and stable body weight throughout the treatment period (Figure S11D). Next, on-target activity of NA was validated by treating 5T33MM-inoculated mice with established disease with 7.5 or 12.5 mpk NA for 5 consecutive days. Western blot analysis of purified 5T33MM cells retrieved from these mice confirmed reduced, albeit not statistically significant, levels of DNMT3B, c-MYC, Aurora kinase B and Cyclin B1, with the most pronounced reduction observed in mice treated with the highest dose. Again, no overt toxicity was observed (Figure S11D-E). Finally, long term NA treatment also increased surface expression of CD38, thereby enhancing the sensitivity of the MM cells to the anti-CD38 moAbs Dara and Isa (Fig. 7G-H, Figure S12A-B).

## Discussion

Increasing evidence is showing that epigenetic reprogramming of MM cells using EMAs holds promise to counter MM progression and relapse upon treatment with SoC agents and emerging immunotherapies, but the knowledge-gap in which epiplayers are key in MM progression and therapy responses and the lack of selective non-toxic EMAs, severely hamper clinical implementation of EMA-based therapies for MM.

In the present study, we show that while somatic DNMT3B mutations appear absent in MM patients [47], *DNMT3B* levels are gradually increasing from the MGUS, over the SMM and ND to the relapse stage. These findings are in line with and extend earlier studies, showing that DNMT3A/B levels are increased in primary MM and PC leukemia cells compared to normal PCs and PCs from MGUS patients [20, 21]. In addition, we show that high *DNMT3B* levels are linked with an aggressive phenotype, as evidenced by particular high *DNMT3B* levels in patients with del17, TP53 mutations or belonging to the PR molecular subgroup; all of which are linked with a bad prognosis. Further underscoring the link with an aggressive phenotype, we observed a clear association between high *DNMT3B* levels and poor outcome in several independent patient cohorts of ND and relapsed patients, irrespective of the therapy applied (monotherapy with Bz, daratumumab or lenalidomide vs. double or triple combinations) and independent of the adverse events del17p or 1q gain. Together, these data highlight the potential of *DNMT3B* as a robust prognostic marker for high risk disease independent from high risk cytogenetic abnormalities and imply a possible role for DNMT3B in MM disease progression and treatment resistance.

While earlier studies have already indirectly suggested an oncogenic role for DNMT3B in MM [20–22], we are the first to establish this formally. Employing two different targeting approaches, genetic depletion and pharmacological inhibition using the DNMT3B selective inhibitor NA, we observed strong anti-myeloma effects, with a strong reduction in cell proliferation and clear induction of apoptosis. Similar findings were documented in T-ALL and Burkitt’s lymphoma, where *DNMT3B* KD and low doses of NA led to a reduction in the number of cells in S-phase, while higher NA concentrations resulted in cell death [18]. Furthermore, in line with its suggested role in cancer cell stemness [5], DNMT3B targeting also strongly reduced MM cell clonogenic outgrowth, which is the ability of a single cell to grow out into a colony. Supporting our findings, a recent study in MM showed that NA reduces the side population and expression of the cancer stem cell core genes Nanog, Oct-4 and Sox-2 in HMCLs [22]. Moreover, a study in melanoma showed that low doses of NA eradicate melanoma cells with clonogenic capacity, whereas higher concentrations are cytotoxic [48]. We made similar observations given that low concentrations of NA and DAC were only able to strongly reduce clonogenic outgrowth, while higher concentrations were also able to eradicate the already established colonies. Of note, it is well-known that the pan-DNMTi AZA and DAC have two modes of action, with low doses resulting in demethylation and epigenetic reprogramming, while high doses are in contrast massively cytotoxic due to the induction of DNA damage [49].

Transcriptomics and GSEA after *DNMT3B* KD further supported the important role for DNMT3B in MM cell cycle progression and stemness, with downregulated gene sets being mainly involved in cell cycle - apoptosis and stemness - maturation related pathways. Western blot analysis furthermore confirmed this on protein level, with c-MYC, Cyclin D1, Cyclin B1, Aurora kinase A (AURKA), AURKB and β-catenin all being significantly down-regulated. MYC is a well-known transcriptional regulator of a multitude of cellular programs, including cell cycle regulation, survival, cell fate decisions, ribosome biogenesis and translation, thus having a tumor-promoting role in cooperation with other oncogenic events in the vast majority of cancers [39, 50, 51]. In MM, deregulated MYC activity, either through amplifications, translocations or altered gene expression, occurs in a large portion of MM cases (± 70%) and is associated with exacerbating disease, poor outcome and emergent DR [38, 52–55]. However, despite many decades of research, pharmacological inhibition of MYC activity remains a major challenge, with no stand-alone treatment specifically targeting MYC being approved at present [39]. In the present study, we show that DNMT3B depletion strongly reduced MYC proteins levels. This is in line with previous studies, where we and others showed that multiple epiplayers, including BET proteins, EZH2, G9a and HDACs, support high c-MYC levels in MM cells by enhancing its transcription and targeting these epiplayers resulted in a concomitant reduction in MYC levels and potent anti-myeloma activity [52, 56, 57]. However, in contrast to the above mentioned epiplayers, DNMT3B targeting did not influence *c-MYC* mRNA levels. In contrast, we show that DNMT3B rather supports high MYC protein levels in MM cells by enhancing MYC protein stability. MYC stability is mainly controlled by its conserved MYC Box I (MBI) region, which contains two key phosphorylation sites, S62 and T58. In response to growth signals, MYC is stabilized through phosphorylation of S62 (pS62) mediated by various kinases involved in pro-survival signalling and cell cycle regulation, including ERK, CDKs, JNK, PLK1 and PIM1. pS62 in turn primes MYC for GSK-3-mediated T58 phosphorylation, which subsequently triggers S62 dephosphorylation by PP2A. pT58 in the absence of pS62 marks MYC for recognition by SCF^Fbw7^, the main E3 ubiquitin ligase for MYC, leading to its proteasomal degradation. In healthy cells, this phosphorylation cycle controls MYC turnover, preventing excessive MYC accumulation. However, in cancers cells, including MM cells, this cycle is disrupted due to the inhibition of PP2A activity or enhanced expression/activity of numerous kinases involved in pro-survival signalling and cell cycle regulation, leading to high pS62 MYC levels and MYC stabilization. Moreover, some of these pro-tumoral kinases can also protect MYC from proteasomal degradation by physically interacting with MYC (AURKA) or by inducing degradation of SCF^Fbw7^ (PLK1 and PKA) [39–41]. Here, we provide strong evidence that DNMT3B protects MYC from proteasomal degradation by preserving high pS62 MYC levels. Additionally, DNMT3B depletion also down-regulated AURKA levels, implying that DNMT3B may also regulate MYC stability by regulating AURKA levels. However, how DNMT3B is regulating these processes remains unclear and requires further investigation. Moreover, the PI MG132 only partially rescued c-MYC’s increased turnover upon DNMT3B depletion, suggesting that DNMT3B may also promote high MYC levels by regulating additional processes, such as MYC translation. Importantly, we further demonstrated that enforced c-MYC (over)expression sensitizes MM cells to both DNMT3B depletion and NA treatment, while DNMT3B mRNA and protein levels were significantly increased in these MYC-overexpressing cells. The latter observation is in line with studies in T-ALL and lymphoma, showing that increased DNMT3B levels are, apart from reduced miR-29a/b levels, also the result of increased MYC levels [18]. On these bases, we speculate that there exists a reinforcing loop between MYC and DNMT3B in MM cells, supporting growth, survival, clonogenicity and stemness related transcriptional programs, and that strong c-MYC activity is required to inflict potent killing in MM cells upon DNMT3B targeting.

The MYC dependence of DNMT3B resulting in synthetic lethality upon DNMT3B targeting is of great importance, as triple-relapsed/refractory and extramedullary tumors were recently shown to upregulate G2/M checkpoint gene sets and E2F and MYC targets along with reduced expression of tumor antigens (CD38, SLAMF7, GPRC5D,…) compared to newly diagnosed MM or relapsed MM pre-Dara exposure [58, 59]. Hence, we hypothesized that DNMT3B targeting might be a good strategy to delay resistance to current SoC agents and emerging immunotherapies. In line with this hypothesis, we observed clear synergistic interaction when NA treatment was combined with Bz or Mel. Moreover, NA was found to resensitize Bz and Mel resistant cells, increase the anti-clonogenic capacity of Bz and Mel and enhance the anti-myeloma activity of Bz in DNMT3B depleted cells and in the syngeneic murine 5T33MM model. Long-term NA treatment was also able to enhance CD38 surface expression in several HMCL thereby sensitizing them to Dara and Isa. Finally, we also validated the potent anti-MM activity of NA on human primary samples, showing higher selectivity towards MM cells than towards BM stromal cells, thus further confirming the therapeutic potential of DNMT3B targeting in MM.

One key question that remains unaddressed is which specific DNMT3B regions and functional interactions are critical for MM cell growth and survival. DNMT3B is a large protein containing multiple structured domains essential for DNA binding and scaffolding interactions with multiple partners, including HDACs, chromatin remodeling enzymes, transcriptional regulators, sumoylation and ubiquitination proteins. Moreover, there are multiple DNMT3B isoforms due to alternative splicing, most of which are catalytically inactive and are thought to regulate the activity of the active isoforms [5]. Hence, further research mapping DNMT3B’s critical regions and functional interactors is needed to fully elucidate how DNMT3B contributes to the pathology.

## Conclusions

In conclusion, we provide evidence that *DNMT3B* is upregulated in relapsed MM patients, correlating with aggressive disease and poor outcome. Additionally, we show that DNMT3B supports MM cell growth and survival, by sustaining cell cycle progression and stemness-related transcriptional programs and stabilizing MYC protein, and DNMT3B targeting displays strong anti-MM activity, sensitizing the MM cells to several SoC agents. Thus, our findings unveil DNMT3B as a novel epigenetic regulator of MYC deregulation in MM and a therapeutic vulnerability in patients with high DNMT3B and MYC levels.

## Electronic supplementary material

Below is the link to the electronic supplementary material.

Supplementary Material 1

Supplementary Table 2

Supplementary Table 3

Supplementary Table 4

## Data Availability

The raw data generated in this study are available upon request.

## Abbreviations

MM: Multiple Myeloma
SOC: Standard Of Care
PIs: Proteasome Inhibitors
IMiDs: Immunomodulatory Drugs
moAbs: monoclonal Antibodies
DR: Drug Resistance
HMTs: Histone Methyltransferases
MMSET: Multiple Myeloma SET domain
EZH2: Enhancer of Zeste 2
HDACi: Histone Deacetylase Inhibitors
DNMTi: DNA Methyltransferase Inhibitors
EMAs: Epigenetic Modulating Agents
AZA: Azacytidine
DAC: Decitabine
Dara: Daratumumab
Isa: Isatuximab
Bsd: Blasticidin
CHX: Cycloheximide
DNMT3B: DNA Methyltransferase 3B
AML: Acute Myeloid Leukemia
T-ALL: T-cell ACUTE LYMPHOBLASTIC LEUKEMIA
HMCLs: Human Myeloma Cell Lines
Bz: Bortezomib
Mel: Melphalan
PR: Proliferation group
CD-1: Cyclin D1 overexpression group
CD-2: Cyclin D3 overexpression
LB: Low Bone disease
HY: Hyperdiploid
GPI: Gene expression-based Proliferation Index
ND: Newly Diagnosed
PC: Plasma Cell
BM: Bone Marrow
PBMC: Peripheral Blood Mononuclear Cells
MGUS: Monoclonal Gammopathy of Undetermined Significance
SMM: Smoldering Myeloma
NA: Nanaomycin A
GSEA: Gene Set Enrichment Analysis
