# Supplementary material for: The de novo DNA methyltransferase 3B is a novel epigenetic regulator of MYC in multiple myeloma, representing a promising therapeutic target to counter relapse
Source: J Exp Clin Cancer Res. 2025 Apr 17;44:125. doi: 10.1186/s13046-025-03382-y (PMC12004749; doi:10.1186/s13046-025-03382-y)
Supplement: Supplementary file 1 — Supplementary Material 1 [file 13046_2025_3382_MOESM1_ESM.docx]

**The *de novo* DNA methyltransferase 3B is a novel epigenetic regulator of MYC in multiple myeloma, representing a** **promising therapeutic target to counter relapse**

Catharina Muylaert^1^, Lien Ann Van Hemelrijck^1^, Arne Van der Vreken^1^, Robbe Heestermans^2-3^, Hatice Satilmis^1^, Emma Verheye^1^, Elina Alaterre^4^, Catharina Olsen^5-7^, Nathan De Beule^1,2^, Kim De Veirman^1,2^, Eline Menu^1^, Karin Vanderkerken^1^, Jérôme Moreaux^8-10^ and Elke De Bruyne^1*^

**Supplemental material and methods**

**Gene expression profiling data**

We used microarray data from normal BMPC, MM cells and HMCLs (E-MTAB-372, E-TABM-1088 and E-TABM-937) to compare DNMT3B levels during disease progression. Moreover, we used microarray data from purified primary MM cells of ND patients of the UAMS TT2 cohort (GSE4581) to compare DNMT3B levels between the different MM molecular subgroups and gene expression-based proliferation index (GPI) subgroups^1^. We also used publicly available RNA-Seq data of the MMRF-CoMMpass study to compare the DNMT3B expression levels in patients harboring a TP53 mutation or not and in patients with a cytogenetic abnormality. Finally, prognostic value of DNMT3B in terms of event free or progression free survival was evaluated in 674 newly diagnosed patients from the CoMMpass study and 173 relapsed patients from the Mulligan cohort (GSE9782) respectively.

**Human myeloma cell lines**

A broad panel of human myeloma cell lines (HMCLs) covering (epi)genetic diversity of patients was used, including both IL-6 independent and dependent cell lines^2-4^. The IL-6 independent cell lines RPMI-8226, AMO-1, OPM-2, JJN3 and U266 were obtained from ATCC (Molsheim Cedex, France) and cultured in RPMI-1640 medium (Gibco - Thermofisher, Waltham, Massachusetts, U.S.) supplemented with 10% fetal calf serum (FCS; pan-biotech, Aidenbach, Germany) and 2 mmol/L L-glutamine (Gibco). The IL-6 dependent cell lines XG-2, XG-7 and XG-11, were obtained as previously described and cultured in RPMI-1640 medium supplemented with 10% FCS, 2 mmol/L L-glutamine and 2 ng/mL recombinant IL-6 (Miltenyi Biotec, Leiden, The Netherlands)^5^. Cell lines were regularly checked for mycoplasma infection and the identity was regularly checked by short-tandem repeat analysis.

**Viability assay**

Effect on viability was examined by the CellTiter-Glo assay (Promega, Leiden, The Netherlands). Briefly, cell suspensions were resuspended before transferring 50 μL to an opaque-walled 96-well plate in triplicate (Promega). Next, 50 μL CellTiter-Glo® 2.0 reagent was added to the cell suspension (1:1 ratio) and the viability was measured after 15 min of incubation at room temperature using the GloMax® Explorer Multimode Microplate Reader (Promega).

**Apoptosis assay**

Apoptosis of the MM cells was determined by flow cytometry (FACSCanto, BD Biosciences, Franklin Lakes, USA). After cell collection, cells were washed twice with phosphate buffered saline (PBS; Thermofisher) and stained with 1 μL AnnexinV-allophycocyanin (APC) and 1 μL 7’aminoactinomycin D (7’AAD) in 100 μL 1x Annexin Binding Buffer (all from BD Bioscience). Cells were incubated for 15 min in the dark after which 300 μL 1x Annexin Binding Buffer was added to each sample and flow cytometric analysis was performed. The obtained FACS data was analysed with FACSDiva software (BD Biosciences).

**Cell cycle analysis**

Effect on cell cycle was determined by flow cytometry using a Bromo-deoxyuridine (BrdU) and a propidium iodide (PI) staining. Briefly, BrdU (1 μg/mL; Sigma-Aldrich, Saint Louis, Missouri, USA) was administered to the cells 4h before harvesting. Next, cells were washed with 500 μL FACS flow and fixed in 250 μL paraformaldehyde (VWR chemicals, Leuven, Belgium) for 10 min at 4°C. Afterwards, 250 μL PBS (Gibco) and 500 μL PBS-T (PBS, Gibco, + 0.2% tween, Sigma-Aldrich) were added to the cells, and they were incubated overnight at room temperature. The next day, cells were washed with 500 μL FACS flow and incubated for 30 min in 250 μL 2M HCl at room temperature. Afterwards, cells were washed first with 500 μL FACS flow, and then with 500 μL PTF buffer (Phosphate Buffered Saline + 0.5% Triton + 10% serum). Next, cells were resuspended in 50 μL PTF and 3 μL anti-BrdU antibody (anti-BrdU-Fluorescein, #11202693001, Sigma-Aldrich or BrdU Monoclonal Antibody (BU20A) APC, # 17-5071-42, eBioscience) and incubated for 30 min in the dark at room temperature. Cells were then washed with 300 μL PTF, centrifuged for 5 min and washed with 500 μL FACS flow. Next, 100 μL of the cell cycle analysis (CCA) staining solution (composed of 100 μg/mL RNase A, 50 μg/mL propidium iodide and 1X tritron NaCitrate solution) was added to the cells. Finally, 300 μL FACS flow was added and the samples were analysed using the FACSCanto and FACSDiva software (BD Bioscience).

**Colony Forming Assay (CFU)**

Incomplete Methocult medium 04230 (Stemcell Technologies, Vancouver, Canada) was aliquoted (2.4 mL) and stored at -20°C. In general, 2.4 mL incomplete Methocult media was supplemented with 330 μL FCS and 270 μL FCS free medium and vortexed thoroughly. Next, 300 μL cell suspension containing 7 500 cells was added. To ensure that all components were mixed well, tubes were again vortexed for 4 seconds. Next, 1.1 mL of the mixture containing 2 500 viable cells was dispensed into each 35 mm dish (SmartDishTM 6-well culture plates, Stemcell Technologies). Treatment was added either on the day of plating (day 0) or on day 7. For the latter, 400 μL of 10% FCS medium or 10% FCS medium containing the treatment was added. All CFU plates were incubated at 37°C in 5% CO2 for 14 days, after which the plates were analysed using the EVOS M7000 (Thermo Fisher Scientific). The number of colonies was counted using ImageJ Software.

**Quantitative real-time PCR**

Total RNA was extracted, purified and quantified in a similar way as for the RNA sequencing. Afterwards, 1 μg RNA was converted into cDNA by using the Verso cDNA Synthesis Kit (Thermofisher) and the Swift™ Maxi Thermal Cycler (ESCO, Hatboro, Pennsylvania, U.S.). Next, 25 ng of cDNA was mixed with 12.5 μL of PowerUp™ SYBR™ Green Master Mix (Thermofisher), 0.5 μL of mixed forward (10 μM) and reverse primer (10 μM) and 7 μL nuclease-free water. Quantitative real-time PCR (qRT-PCR) was then performed with the Quant Studio 12K Flex System (Thermofisher). To quantify the relative target gene expression, a standard curve was made. ABL was used as reference gene. Primer sequences were as follow (5’-3’): human DNMT3B: forward: GGAGCCACGTAACAAATA; reverse: GTAAACTCTAGGCATCCGTCATC; human DNMT1: forward: CGGCCTCATCGAGAAGAATATC; reverse: TGCCATTAACACCACCTTCA; human DNMT3A: forward: CTGAGGTAGCGACACAAAGTTA; reverse: CTCTTCTGGGTGCTGATACTTC; human c-MYC: forward: CTACCCTCTCAACGACAGCA; reverse: TCATCTTCTTGTTCCTCCTCAGA; human ABL: forward: GAGGGCCTGTGGAAGAAATA; reverse: CACCAGGTTAGGGTGTTTGA. All primers were purchased from Integrated DNA Technologies (IDT, Leuven, Belgium).

**Western blot**

Cell pellets were lysed for 10 min on ice with lysis buffer containing 50 mM Tris, 150 mM NaCl, 1% Nonidet P40 and 0.25% sodium deoxycholate. To inhibit proteases and phosphatases, 4 mM Na3O4, 1 mM Na4P2O7 2 μg/mL aprotinin, 50 μg/mL leupeptin, 500 μg/mL trypsin inhibitor, 10 μM benzamidine, 2.5 mM para-nitrophenyl benzoate (all from Sigma-Aldrich), 50 mM NaF, 5 mM methylenadiaminetetraacetic acid (both from VWR International), 1 mM 4-(2-aminoethyl) benzene sulfonyl fluoride hydrochloride and 50 μg/mL pepstatin A (both from ICN) were added. Next, samples were sonicated using the Bioruptor Pico (Diagenode, Seraing, Belgium) using four cycles of 30 sec on - 30 sec off on 4°C. Afterwards, cell debris was removed by centrifugation (5 min, 14 000 rpm) and protein samples were mixed 1/1 with loading buffer (LB) composed of β-mercaptoethanol and Laemmli sample buffer (Bio-Rad, Temse, Belgium). After the samples were cooked for 5 min, proteins were separated by sodium dodecyl sulfatepolyacrylamide gel electrophoresis (SDS-PAGE). Next, samples were transferred to a PVDF (polyvinylidene fluoride) membrane (Bio-Rad, Temse, Belgium) and membranes were blocked for one hour in Tris-buffered saline (TBS) blocking buffer, containing 5% low-fat milk and 0.1% Tween 20. After blocking, membranes were incubated overnight with primary antibodies (Abs) and two hours with the secondary Ab. Visualization of the proteins was performed with the Li-Cor Odyssey Fc (Li-Cor, Bad Homburg, Germany) after adding Western Lighting Plus enhanced chemiluminescence (ECL) substrate (Perkin Elmer, Waltham, Massachusetts, U.S.) or Supersignaling West Pico PLUS chemiluminescent substrate (Thermofisher). Further analysis was performed with the Image Studio Lite software. Primary Abs used were: DNMT1 (#5032), human DNMT3B (#57868), mouse DNMT3B (#44145), c-MYC (#5605), L-MYC (#76266), pS62MYC (#13748), Cyclin B1 (#4138), Aurora Kinase A (#3092), Aurora Kinase B (#3094), p27 (#3688), β-catenin (#8480), ubiquitin (#3936), α-tubulin (#2144) and β-actin (#4967) from Cell Signaling Technology (Leiden, The Netherlands) and Cyclin D1 (sc-8396) from Santa Cruz Technologies. Anti-rabbit IgG HRP-linked Ab (#7074) from Cell Signaling Technology (Leiden, The Netherlands) was used as secondary Ab.

**Immunoprecipitation**

Cells were collected and resuspended in 1x cell lysis buffer (CLB; Cell Signaling) containing 1 mM phenylmethylsulfonyl fluoride (PMSF; Sigma-Aldrich). After 10 min of incubation on ice, samples were centrifuged for 10 min at 14 000 rpm at 4°C. To reduce nonspecific binding, cell lysates were then first pre-cleared with pre-washed G protein magnetic beads (Cell Signaling). Next, 500 µg of total protein per sample was incubated overnight at 4°C with rotation with c-MYC (#5605) or Rabbit (DA1E) mAb IgG isotype control (#3900S). Next, the lysates containing the primary antibodies were transferred to pre-washed beads and incubated for 30 min on a shaker at room temperature. After incubation, the beads were washed three times with 500 µL of 1x CLB and resuspended in 20 µL of LB. From this point, the samples were prepared for Western blot analysis as described above. The Pierce™ Clean-Blot™ IP Detection Kit (Thermofisher) was used for visualisation of the proteins.

**Drug combination studies**

The interaction between NA and either Bz or Mel was examined using a concentration matrix test, in which every single one of the increasing doses of NA was combined with every single one of the increasing doses of either Bz or Mel. After four days of treatment, viability was tested using the CellTiter Glo method. Next, synergy scores were calculated based on the Bliss method, following the Bliss equitation: 𝑓𝑢C = 𝑓𝑢𝐴.𝑓𝑢B as previously described^6^. Positive values higher than 10 indicate significant synergism, while negative values lower than -10 indicate significant antagonism.

**Patient samples**

BM samples were collected for routine diagnostic or evaluation purposes after patients’ written informed consent was given and in accordance with the Declaration of Helsinki and institutional research board approval from Brussels University hospital (B.U.N. 143201838414) and Montpellier University hospital (DC2008-417). Isolation of the mononuclear cells was performed using density gradient centrifugation with Lymphoprep™ (STEMCELL™ technologies, Grenoble, France). For the total BM experiments, isolated mononuclear cells were treated for four days with NA after which viability and cell counts were assessed as well as the percentage of CD138+ viable plasma cells and CD138- viable non-myeloma cells by flow cytometry using CD138-PE Ab (Beckman Coulter, Indianapolis, United States). For the experiments with purified MM cells and BMSC, a MACS separation using human CD138+ MicroBeads (Miltenyi Biotec) was performed according to manufacturer’s instructions. The positive fraction containing the MM cells was cultured and treated up to 48h in RPMI medium containing 10% FCS and 2 mmol/L L-glutamine, while the negative fraction containing the BMSCs was placed in culture in DMEM medium with 10% horse serum, 10% FCS and supplements containing 1% natriumpyruvate, 1% minimum essential medium, 100 U/mL penicillin/streptomycin and 2 mM L-glutamine. One day after plating, the non-adherent cells were removed and the human BMSC were kept in culture until passage 4. After 48h or 4 days of NA treatment, CellTiter-Glo was performed to assess the viability of either MM or BMSC respectively.

**NK purification from PBMC**

NK cells were isolated from the blood of healthy donors. PBMCs were first isolated as described above and NK cells were then isolated by MACS cell separation using CD56+ magnetic beads (Miltenyi Biotec, Bergisch Gladbach, Germany) as described by the manufacturer’s protocol. NK purity was evaluated by flow cytometry using 4 µL of anti-human CD56 PE staining in 100 μL 1x Annexin Binding Buffer (BD Biosciences). NK cells were allowed to rest overnight in RPMI-1640 medium supplemented with 10% fetal calf serum, 2 mmol/L L-glutamine and 4 units/µL IL15 (Miltenyi Biotec).

**RNAscope**

Cells (two million) were washed in 5 mL PBS and centrifuged for 5 min at 300 g. Next, the cell pellet was resuspended in pre-warmed formol and incubated for 30 min at 37°C. Cells were then centrifuged again for 5 min at 300 g and, resuspended in 70% ethanol and spotted (400 000 cells/slide) on superfrost Plus Gold Adhesion Microscope Slides (Epredia, Kalamazoo, Michigan) The slides were left to dry for 20 minutes, after which they were incubated for 5 min in a 50%, 70%, 100% and 100% ethanol solution. Slides were then either stored in the 100% ethanol solution at -20°C or immediately further processed. In case of the latter, slides were baked for 1 h at 60°C, incubated two times for 5 min in xylene, followed by two times a 2 min incubation in 99% ethanol. To allow the slides to dry, they were placed in front of a ventilator for 5 min, incubated for 10 min in a 3% hydrogen peroxide solution (Sigma-Aldrich) to inhibit the endogenous peroxidase activity and subsequently washed 2 times in mQ water. The slides were then put in a bath of RNAscope® Target Retrieval Reagents (ACD, Abingdon, United Kingdon) at 98°C for 15 min, washed again in mQ, immersed for 3 min in 100% ethanol and dried for 5 min. Using the ImmEdge® Hydrophobic Barrier PAP Pen (Vector laboratories, Newark, United States), a barrier was then created around the samples and the samples were incubated with RNAscope Protease Plus (ACD) for 15 min at 40°C using the HybEZ oven (ACD). After another wash in mQ, hybridization was performed by adding either the RNAscope™ 3-plex Positive Control Probe- Hs against POLR2A and PPIB, the RNAscope® 3-plex Negative Control Probe against dapB or the target probes against SDC1 (CD138) and DNMT3B (all from ACD) for 2h at 40°C. After hybridization, the slides were washed 3 times for 5 min in RNAscope wash buffer and kept overnight in saline-sodium citrate (SSC) buffer (Sigma-Aldrich). The next day, slides were again washed 3 times for 5 min and incubated for 30 min with RNAscope Multiplex FL v2 Amp1, 30 min with RNAscope Multiplex FL v2 Amp2 and 30 min with RNAscope Multiplex FL v2 Amp3 with 2-3 washing steps of 5 min in between (ACD). To develop the HRP-C1 signal, RNAscope Multiplex FL v2 HRP-C1 was added for 15 min followed by 2 washing steps of 2 min. Next, TSA Vivid Fluorophore 570 diluted 1:1500 in TSA buffer was added for 30 min and the slides were washed again. Lastly, RNAscope Multiplex FL v2 HRP blocker was added for 15 min, again followed by 2 washing steps of 2 min (ACD). The same procedure was followed for the second fluorophore, using respectively RNAscope Multiplex FL v2 HRP-C2, Opal 690 Fluorophore and RNAscope Multiplex FL v2 HRP blocker (ACD). Then, the slides were incubated for 10 min at room temperature in Hoechst 1:500 in PBS. Next, approximately 1 drop/section of ProLong Gold Antifade Mountant and a high precision glass coverslip (number 1.5) was mounted on the slide and dried overnight in the dark at room temperature. Slides were scanned using the ZEISS Axioscan 7 (Carl Zeiss, Zaventem, Belgium) and analysed using the HALO software (Indica labs, Albuquerque, United States). The Cy3 channel was used for the detection of the TSA Vivid Fluorophore 570 and the Cy5 channel was used for the detection of the Opal 690 Fluorophore.

**Antibody dependent cellular cytotoxicity (ADCC) assay**

Nine days after treatment with NA, cells were labelled with 0.1 µM CellTrace™ CFSE (ThermoFisher) in pre-warmed PBS for 13 min at 37°C. Next, pre-warmed complete medium was added in excess for 20 to 30 min at RT. After this incubation, cells were treated with 1 µg/mL daratumumab or 10 ng/mL isatuximab for 20 min at 37°C. Next, purified NK cells were added with an effector-to-target ratio of 5:1 for 4 hours at 37°C. After 4 hours, cell death was assessed by staining the samples with 1 µL of 7-AAD in 100 μL 1x Annexin Binding Buffer (BD Biosciences) and performing flow cytometric analysis.

**Murine cells**

5T33MMvv cells were purified from terminally diseased mice. Briefly, BM cells were obtained by flushing the tibia and femurs and crushing the vertebrae of the diseased MM mice. Next, cells were treated for 2 minutes with ammoniumchloride to eliminate the red blood cells. To further purify the 5T33vv cells, CD11b MicroBead depletion was performed according to the manufacturer’s instructions (Miltenyi Biotec). 5T33vv cells were cultured in RPMI-1640 medium supplemented with 10% FCS, 1% natriumpyruvate, 1% minimum essential medium, 100 U/ml penicillin/streptomycin and 2 mM L-glutamine (all from Gibco), while murine 5T33BMSC were cultured in DMEM medium with 10% horse serum, 10% FCS and supplements containing 1% natriumpyruvate, 1% minimum essential medium, 100 U/ml penicillin/streptomycin and 2 mM L-glutamine. One day after plating, the non-adherent cells were removed. Murine BMSC were kept in culture until passage 4. Viability was assessed after 24h and 48h for the 5T33vv and after 4 days for the 5T33BMSC upon NA treatment using CellTiter-Glo.

**Mice experiment**

C57BL/KalwRij mice were purchased from Envigo (Horst, the Netherlands) and housed and treated following conditions approved by the Ethical committee (Licence No LA1230281, CEP No 23-281-13). On day 0, 5x10^5^ 5T33 MM cells in 200 µL medium without serum were intravenously injected in naïve C57BL/KalwRij mice. For the first experiment, starting from day 2 post-tumor inoculation, mice were treated 5 times a week with a suboptimal dose of NA (7.5 mg/kg) and 2 times a week with a suboptimal dose of Bz (0.5 mg/kg) or a combination of both compounds. Mice were killed at day 21, when MM disease had reached end-stage. Spleen weight was measured and serum protein electrophoresis was used to evaluate the M-spike. Total bone marrow was isolated and processed as described above and myeloma cells were stained with an in-house made 3H2 anti-idiotype antibody. The % of positive 3H2 myeloma cells was assessed using flow cytometry. For the second experiment, mice with established disease (14 days post-tumor inoculation) were treated for 5 consecutive days with 7.5 or 12.5 mg/kg NA and tumor cells were collected for WB analysis as described before.

**Supplemental Figures**

**
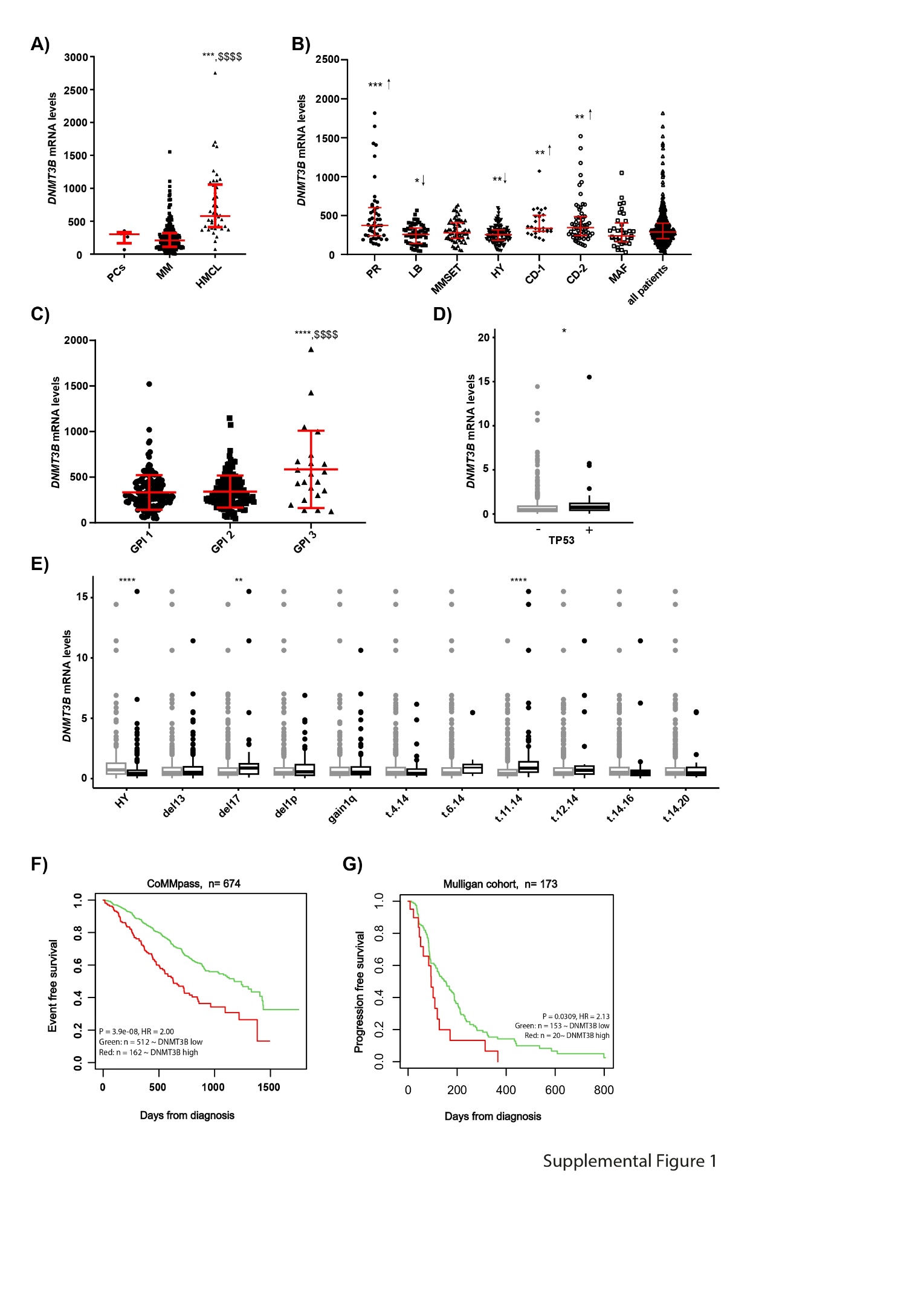
**

**Supplemental Figure 1:** **Expression and prognostic value of *DNMT3B* in terms of event/progression free survival in MM. A)** *DNMT3B* mRNA levels in normal bone marrow plasma cells (PCs, n=5, E-MTAB-372), primary MM cells (MM, n=206, E-MTAB-372) and HMCLs (n=42, E-TABM-1088 and E-TABM-937). *** indicates p≤0.001 compared to PCs, $$$$ indicates p ≤ 0.0001 compared to MM cells. **B)** *DNMT3B* levels in the different molecular MM subsets (GSE4581). * Indicates p≤0.05, ** indicates p≤0.005, *** indicates p≤0.001 compared to all MM patients. PR: proliferation, LB: low bone disease, MMSET: multiple myeloma SET domain, HY: hyperdiploidy, CD-1: *cyclin D1* overexpression, CD-2: *cyclin D3* overexpression and MAF: MAF and MAFB group. **C)** *DNMT3B* levels in the different gene-expression proliferation index (GPI) groups calculated for the TT2 cohort (GSE4581). The GPI score is calculated for each patient based on the expression value of 50 genes linked with proliferation. Low proliferation is indicated as GPI^low^ (GPI1), median proliferation as GPI^median^ (GPI2) and high proliferation as GPI^high^ (GPI3). **** indicates p≤0.0001 compared to GPI1 and $$$$ indicates p≤0.0001 compared to GPI2. **D)** *DNMT3B* levels in patients from the CoMMpass study without (light grey) or with (dark grey) a TP53 mutation. * Indicates p≤0.05 compared to MM patients without TP53 mutation. **E)** *DNMT3B* levels in patients from the CoMMpass study without (light grey) or with (dark grey) the indicated cytogenetic abnormality. ** indicates p≤0.005, **** indicates p≤0.0001 compared to patients not carrying the cytogenetic abnormality. **F-G)** Prognostic potential of *DNMT3B* levels in terms of event or progression free survival in newly diagnosed (ND) (CoMMpass, F) and relapsed MM (GSE9782, G) patients. Maxstat analysis was used to calculate the optimal separation of patients based on a cut-off value.


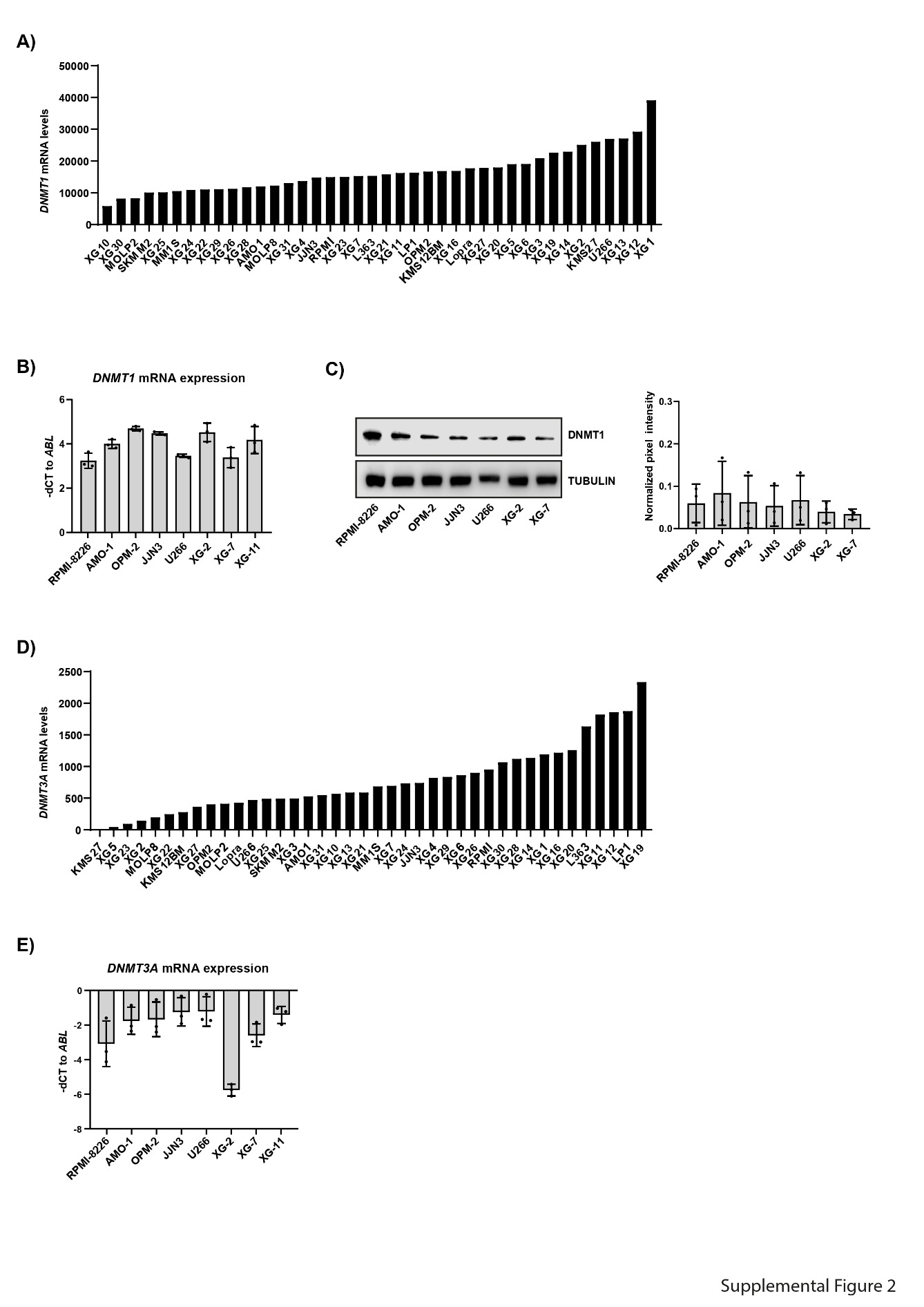


**Supplemental Figure 2: DNMT1 and DNMT3A expression in human MM cell lines.** **A)** *DNMT1* levels in 40 different HMCLs using our own RNA-seq data. **B)** *DNMT1* expression was determined in a selected panel of HMCLs using qRT-PCR. *ABL* was used as reference gene. The mean expression ± SD of three independent experiments is shown. **C)** DNMT1 protein expression determined in a selected panel of HMCLs via western blot. Tubulin was used as loading control. Left: blots of one experiment representative of three are shown, right: quantification of the DNMT1 levels relative to tubulin as measured by Image Studio for the 3 independent experiments. **D)** *DNMT3A* levels in 40 different HMCLs using our own RNA-seq data. **E)** *DNMT3A* expression was determined in a selected panel of HMCLs using qRT-PCR. *ABL* was used as reference gene. The mean expression ± SD of three independent experiments is shown.


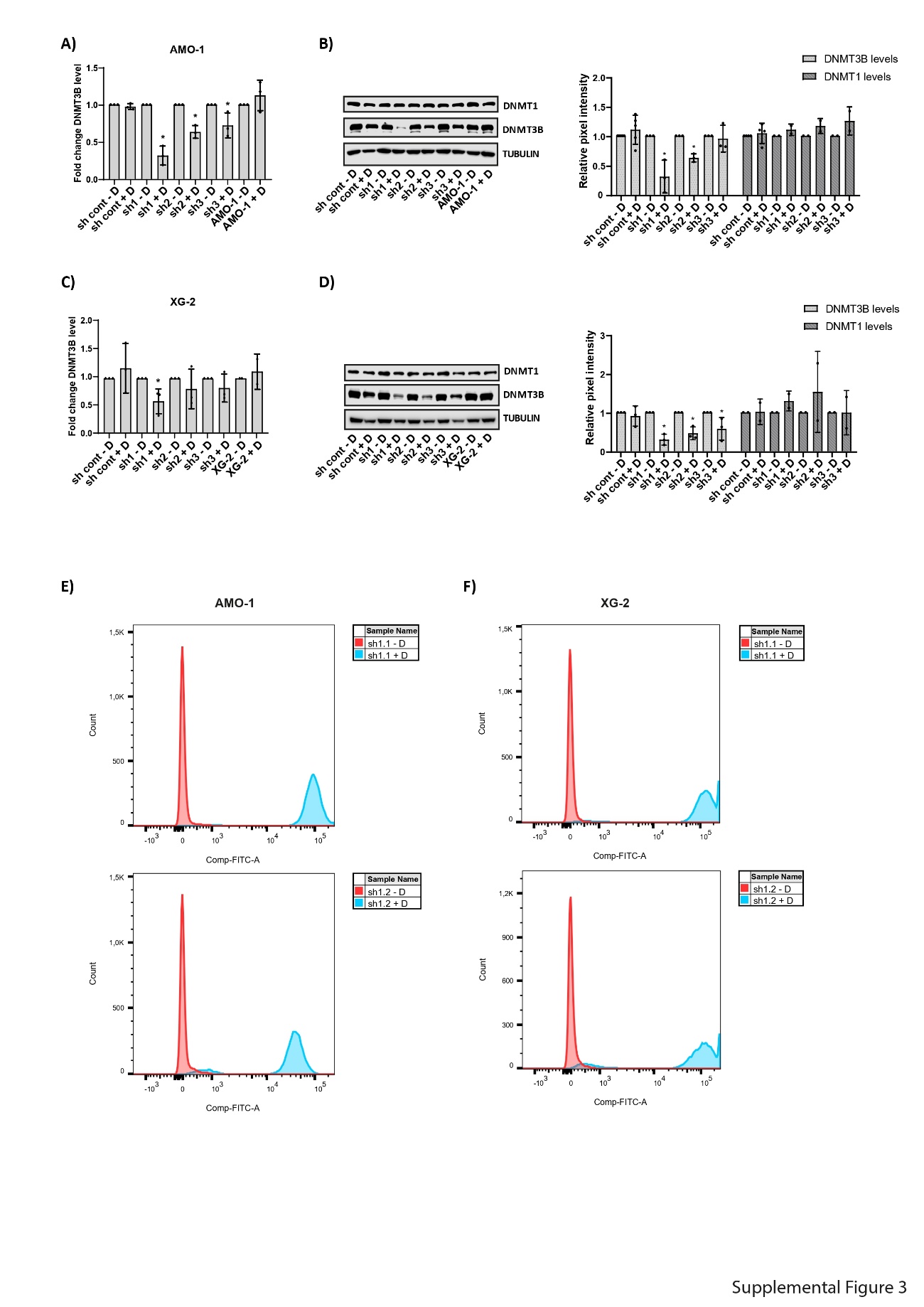


**Supplemental Figure 3: Validation of *DNMT3B* knockdown in AMO-1 and XG-2 HMCL and eGFP histograms of obtained subclones. A-D)** DNMT3B mRNA and protein levels upon *DNMT3B* targeting in *shDNMT3B* nr1 (sh1), *shDNMT3B* nr2 (sh2) or *shDNMT3B* nr3 (sh3) transduced AMO-1 (A-B) and XG-2 (C-D) cell lines. The *DNMT3B* mRNA levels were determined in the AMO-1 (A) and XG-2 (C) transduced cells after 3 days of doxycycline treatment with qRT-PCR. *ABL* was used as reference gene. Relative expression levels in stimulated (+D) compared to unstimulated (-D) cells is shown (n=3). * Indicates p≤0.05 compared to unstimulated cells. The DNMT1 and DNMT3B protein levels in transduced AMO-1 (B) and XG-2 (D) cells were determined by western blot 5 days post-doxycycline treatment. Tubulin was used as loading control. Left panel: one experiment representative of at least three is shown for DNMT3B and one experiment representative of two is shown for DNMT1, right panel: quantification of DNMT1 and DNMT3B levels relative to tubulin as measured by Image Studio and normalized to unstimulated cells. * Indicates p≤0.05 compared to unstimulated cells. **E-F)** eGFP histograms of obtained AMO-1 (E) and XG-2 (F) subclones upon 3 days of doxycycline treatment. One experiment representative of at least three is shown.


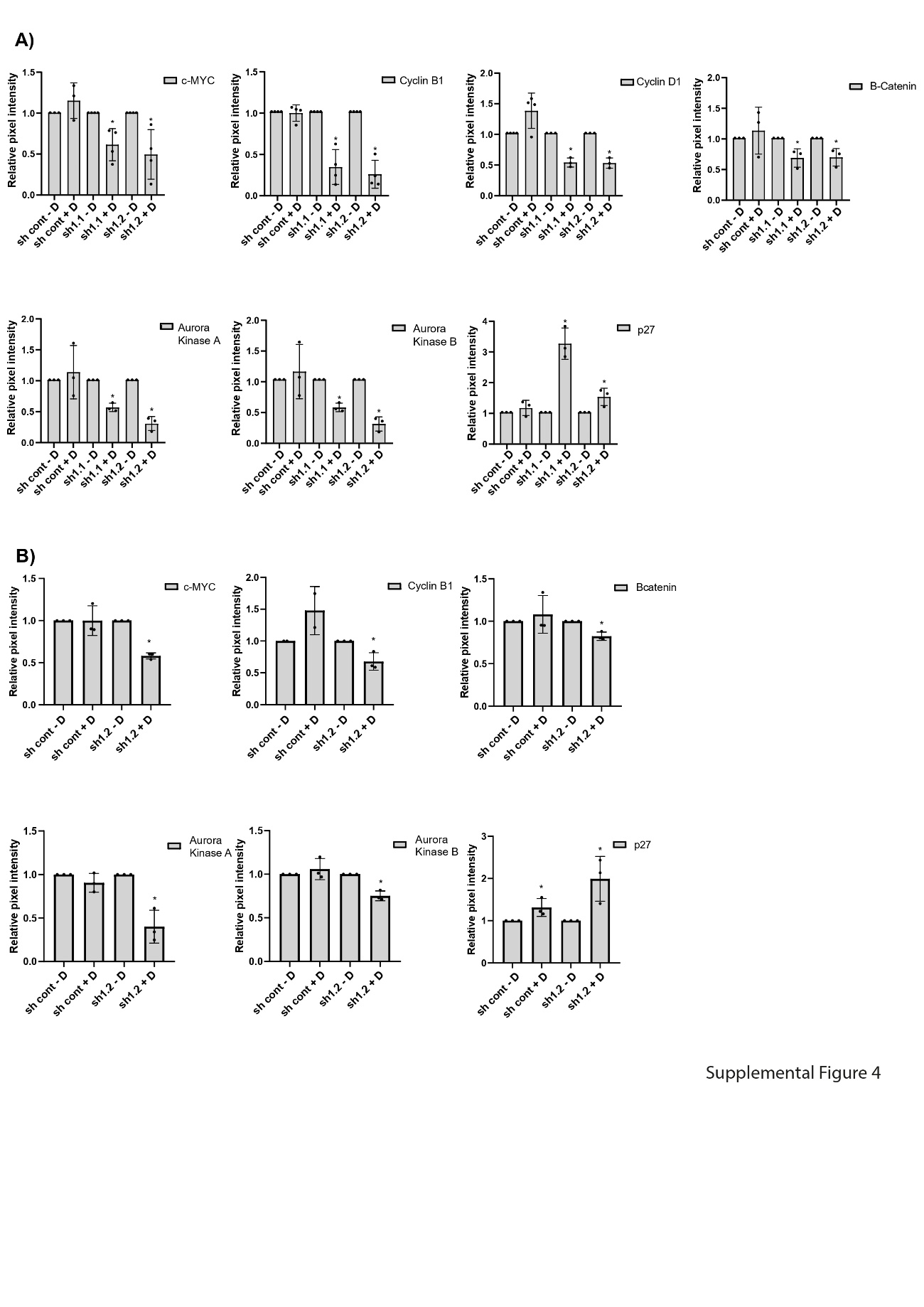


**Supplemental Figure 4: Quantification of western blot data shown in Figure 4D-E for *shDNMT3B* XG-2 and AMO-1 cells.** **A-B)** Pixel density of the bands obtained for c-MYC, Cyclin B1, Cyclin D1, Aurora kinase A, Aurora kinase B, p27 and β-catenin relative to tubulin or actin as measured by Image Studio and normalized to unstimulated (-D) XG-2 (A) or AMO-1 (B) cells. The mean ± SD of at least three independent experiments is shown. * Indicates p≤0.05 compared to unstimulated (-D) cells.


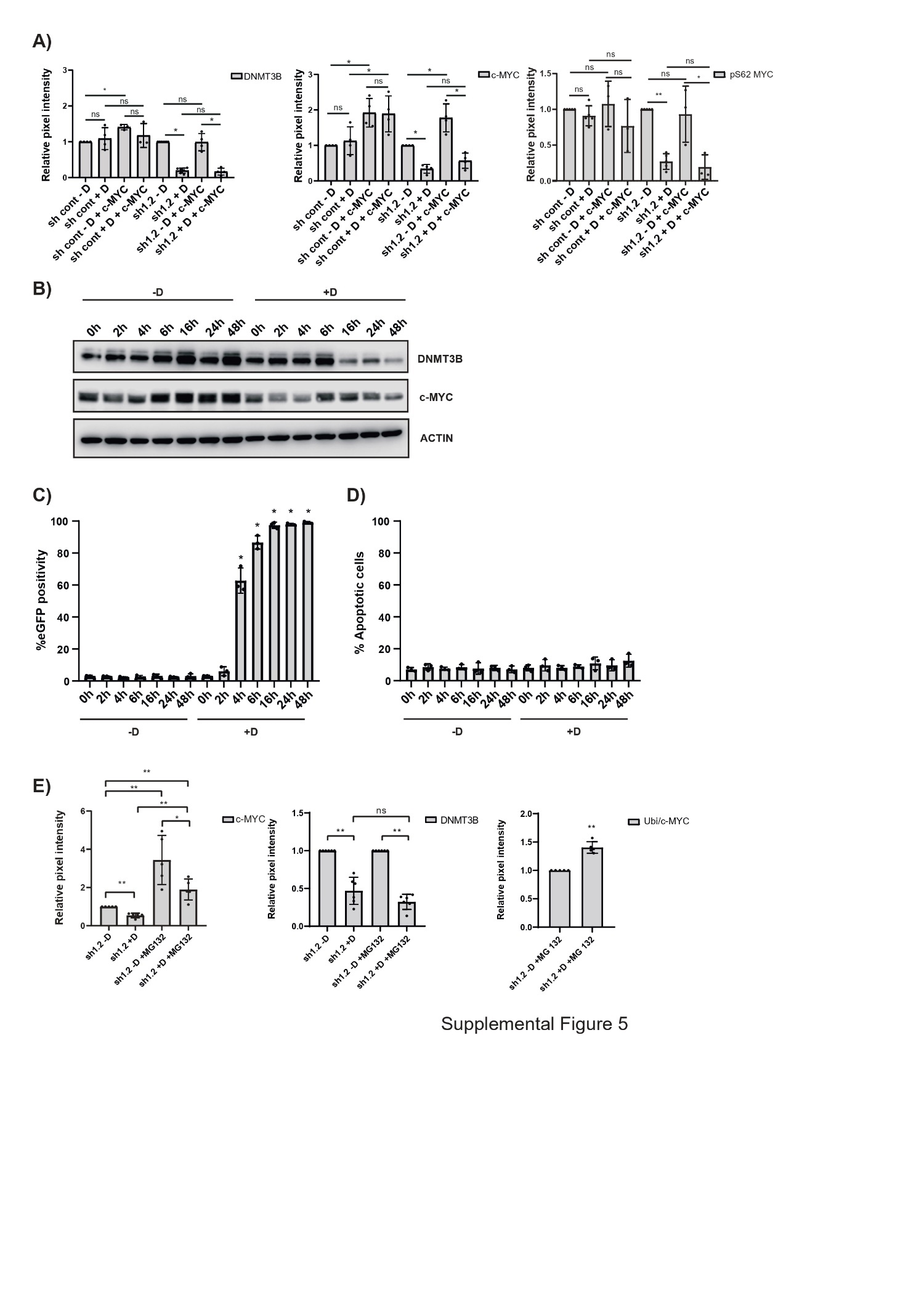


**Supplemental Figure 5: Time kinetics study upon *DNMT3B* KD.** **A)** Relative pixel density of the bands obtained for DNMT3B, c-MYC and pS62MYC relative to actin as measured by Image Studio and normalized to unstimulated (-D) sh control or sh1.2 cells. **B)** XG-2 *shDNMT3B* cells were treated with (+D) or without (-D) doxycycline for indicated timepoints and the protein levels of DNMT3B and c-MYC were followed-up in time by western blot. Actin was used as loading control. One experiment representative of two is shown. **C-D)** XG-2 *shDNMT3B* cells were treated with (+D) or without (-D) doxycycline for indicated timepoints and the % eGFP positive (B) and % apoptotic cells (C) were followed-up in time by flow cytometry. The % apoptotic cells are the sum of AnnexinV (+) and AnnexinV (+)/7’AAD (+) cells. Bars are the mean ± SD of 3 independent experiments. * Indicates p≤0.05. **E)** Relative pixel density of the bands obtained for DNMT3B and c-MYC relative to actin (left and middle) and the ubiquitinated/total c-MYC ratio (right) as measured by Image Studio and normalized to unstimulated (-D) sh1.2 cells.

**
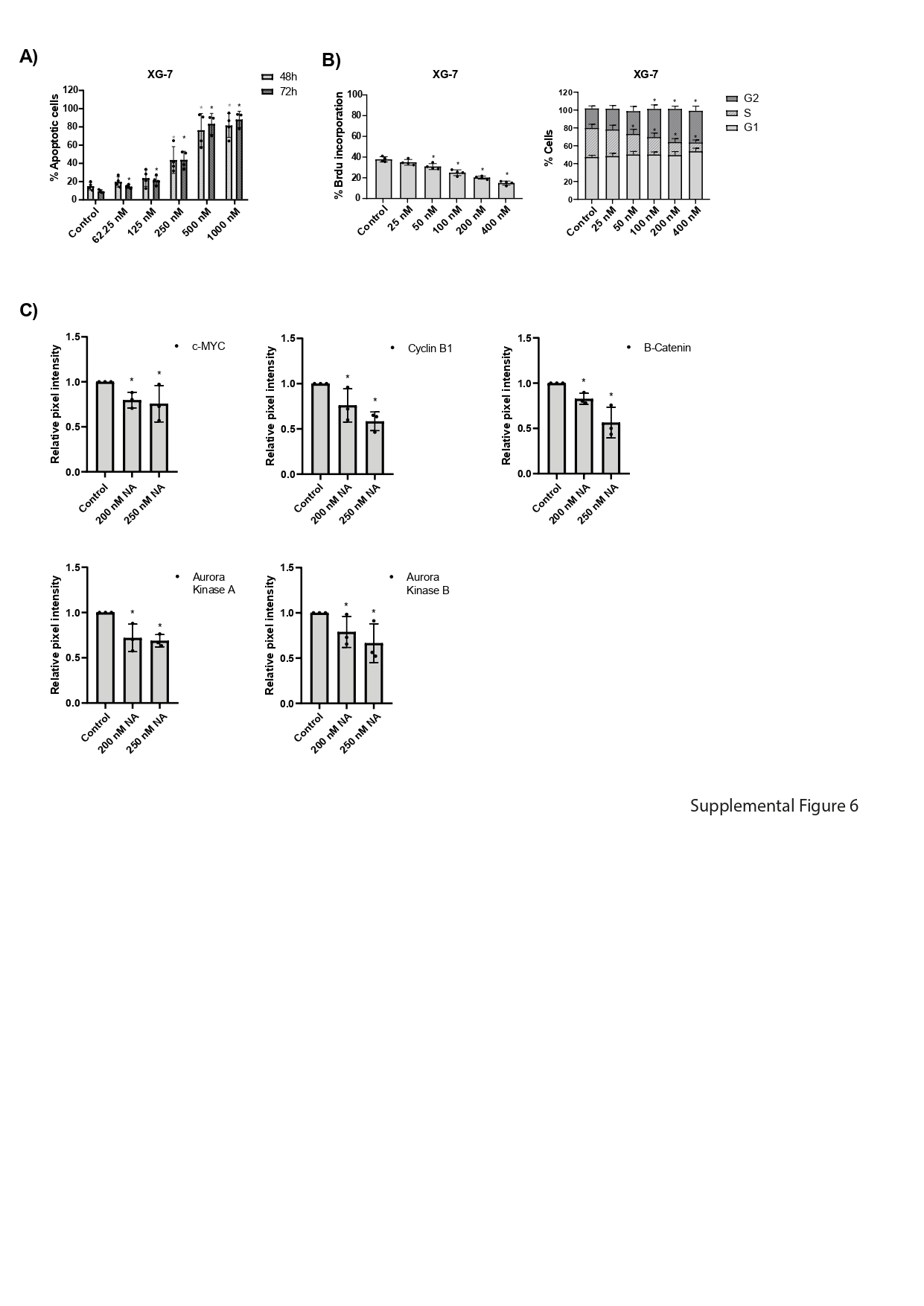
**

**Supplemental Figure 6:** **Effect of NA treatment on HMCL. A)** Effect of NA on cell apoptosis for XG-7. Cells were treated with indicated doses of NA for 48h (light gray bars) and 72h (dark grey bars) and the effect on apoptosis was evaluated using an AnnexinV/7’AAD staining followed by flow cytometric analysis respectively. The % apoptotic cells are the sum of AnnexinV (+) and AnnexinV (+)/7’AAD (+) cells. * Indicates p≤0.05 compared to control. **B)** Effect of NA on MM cell proliferation. Cells were treated with indicated doses of NA for 24h after which the effect on BrdU incorporation (left) and cell cycle progression (right) was determined using BrdU and PI-stainings respectively. * Indicates p≤0.05 compared to control. **C)** Quantification of the western blot data shown in Figure 6E. Pixel density of the bands obtained for c-MYC, Cyclin B1, Aurora kinase A, Aurora kinase B, and β-catenin relative to actin as measured by Image Studio and normalized to control. The mean ± SD of at least three independent experiments is shown. * Indicates p≤0.05 compared to control.


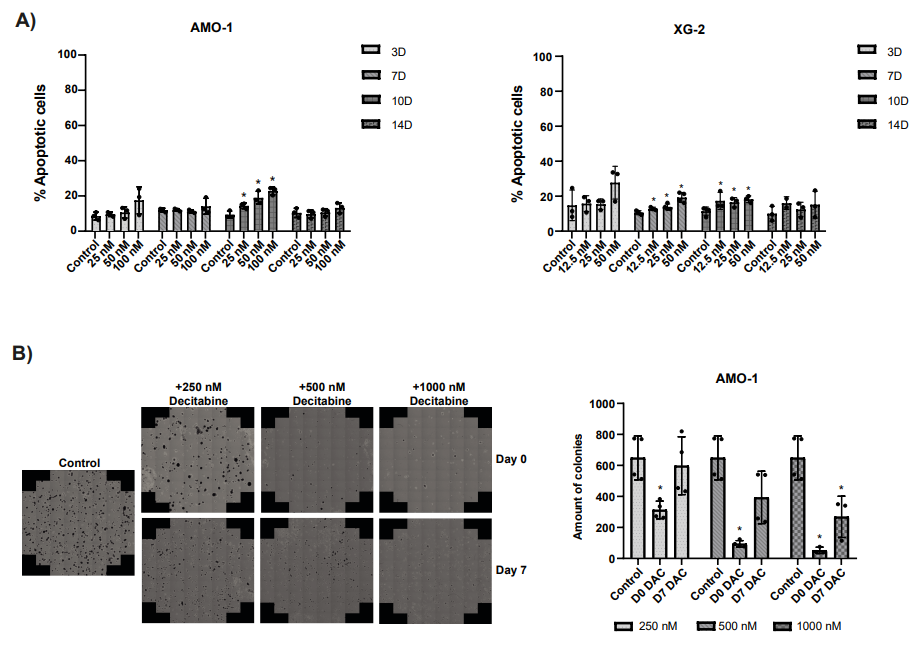
**Supplemental Figure 7: Effect of long-term, low dose Nanaomycin A treatment on apoptosis and effect of decitabine on clonogenic outgrowth. A)** Apoptosis was assessed in the AMO-1 and XG-2 cell lines 3, 7, 10 and 14 days after treatment with indicated low doses of NA. The % apoptotic cells are the sum of AnnexinV (+) and AnnexinV (+)/7’AAD (+) cells. The mean ± SD of at least three independent experiments is shown. * Indicates p≤0.05 compared to control. **B)** Effect of decitabine treatment on MM cell clonogenicity. AMO-1 cells were treated with 250 and 500 nM (low doses) or 1000 nM (high dose) of decitabine on the day of plating (day 0) or 7 days post-plating (day 7). The number of colonies were determined 14 days post-plating using the EVOS M7000 Imaging System. Left: visual representation of the AMO-1 colony forming assay, right: the number of colonies counted with ImageJ software. The mean ± SD of at least three independent experiments is shown. * Indicates p≤0.05 compared to control.

**
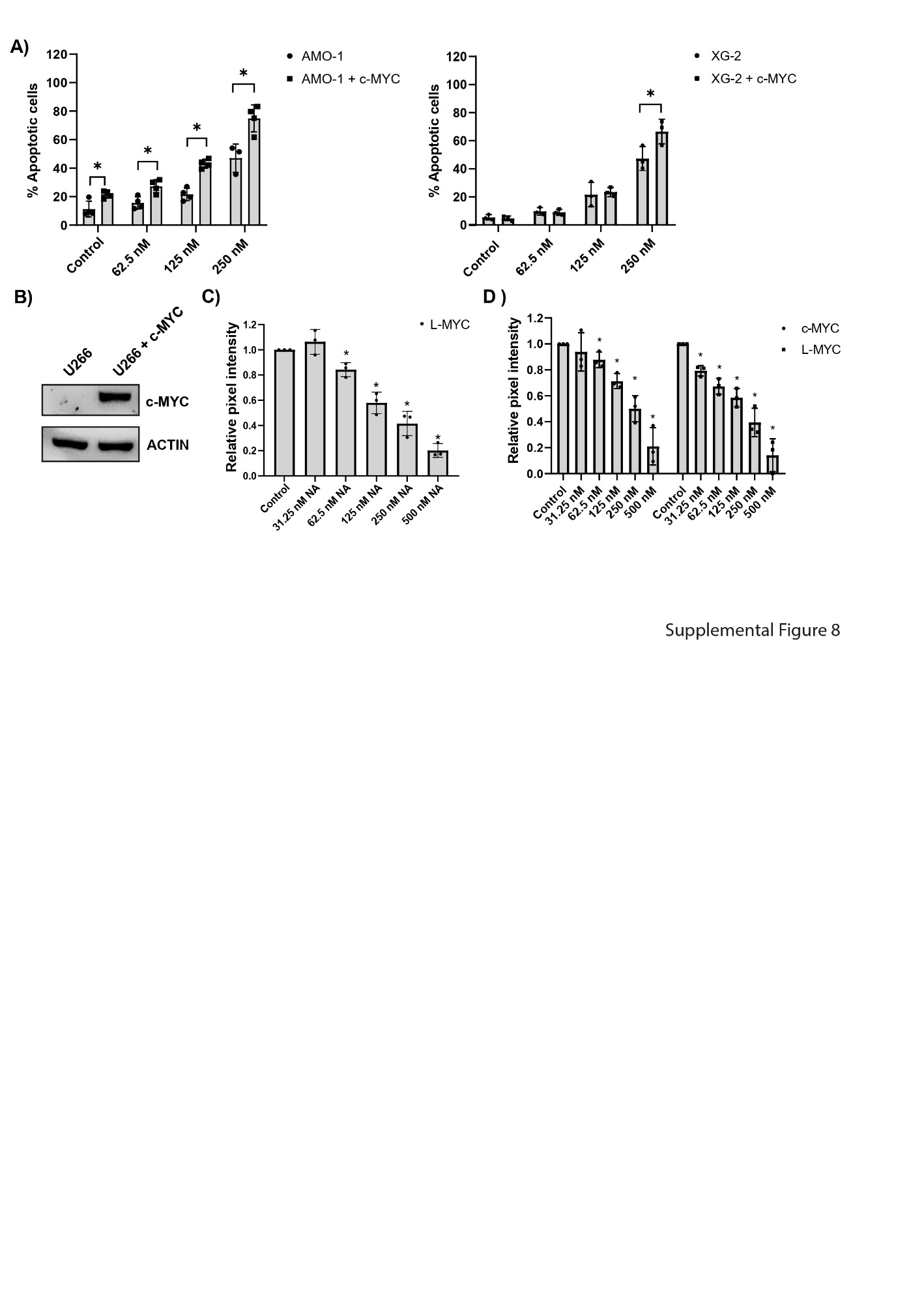
**

**Supplemental Figure 8: Effect of NA treatment on c-MYC overexpressing HMCL. A)** Effect of 3 days NA treatment on apoptosis for the AMO-1 and XG-2 cell lines with or without c-MYC overexpression. The % apoptotic cells are the sum of AnnexinV (+) and AnnexinV (+)/7’AAD (+) cells. The mean ± SD of at least three independent experiments is shown. * Indicates p≤0.05 compared to control. **B)** Validation of c-MYC overexpression in the U266 transduced cells. Actin was used as loading control. One experiment representative of four is shown. **C-D)** Pixel density of the bands obtained for L-MYC in U266 cells (C) and L-MYC and c-MYC in U266 c-MYC overexpressing cells (D) relative to actin as measured by Image Studio and normalized to control. The mean ± SD of three independent experiments is shown. * Indicates p≤0.05 compared to control.

**
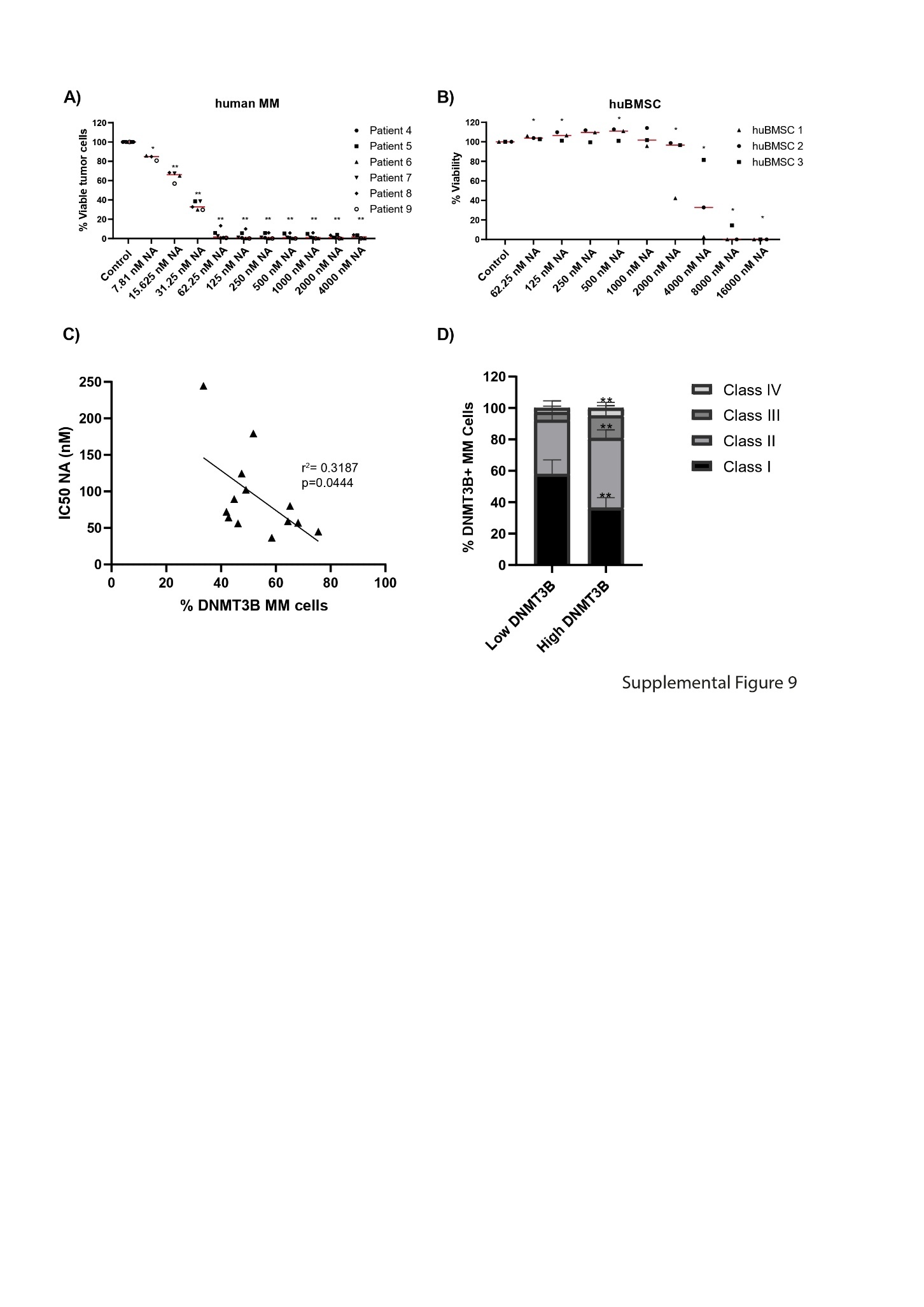
**

**Supplemental Figure 9:** **Effect of Nanaomycin A on primary human MM cells and BMSC.** **A-B)** Effect of NA treatment on the viability of purified human CD138+ MM cells (A) and human CD138- BMSC (B). The viability was assessed after 48h or 4 days of NA treatment respectively using the CellTiter-Glo Luminescent Cell viability assay. The individual measured values, in black, and the median, in red, are shown. * Indicates p≤0.05, ** indicates p<0.01 compared to control. **C)** Inverse correlation between the measured IC50 value after NA treatment and the percentage DNMT3B positive primary human myeloma cells (CD138+) evaluated using RNAscope technology (r^2^= 0.3187 and p=0.044). **D)** Myeloma samples were divided in either the ‘DNMT3B low’ or ‘DNMT3B high’ group and the DNMT3B positive CD138 cells were classified in different classes according to the number of DNMT3B copies present, with class I having only one copy, class II 2 to 4 copies, class III 5 to 9 copies and class IV at least 10 copies. ** indicates p<0.01 compared to low DNMT3B.


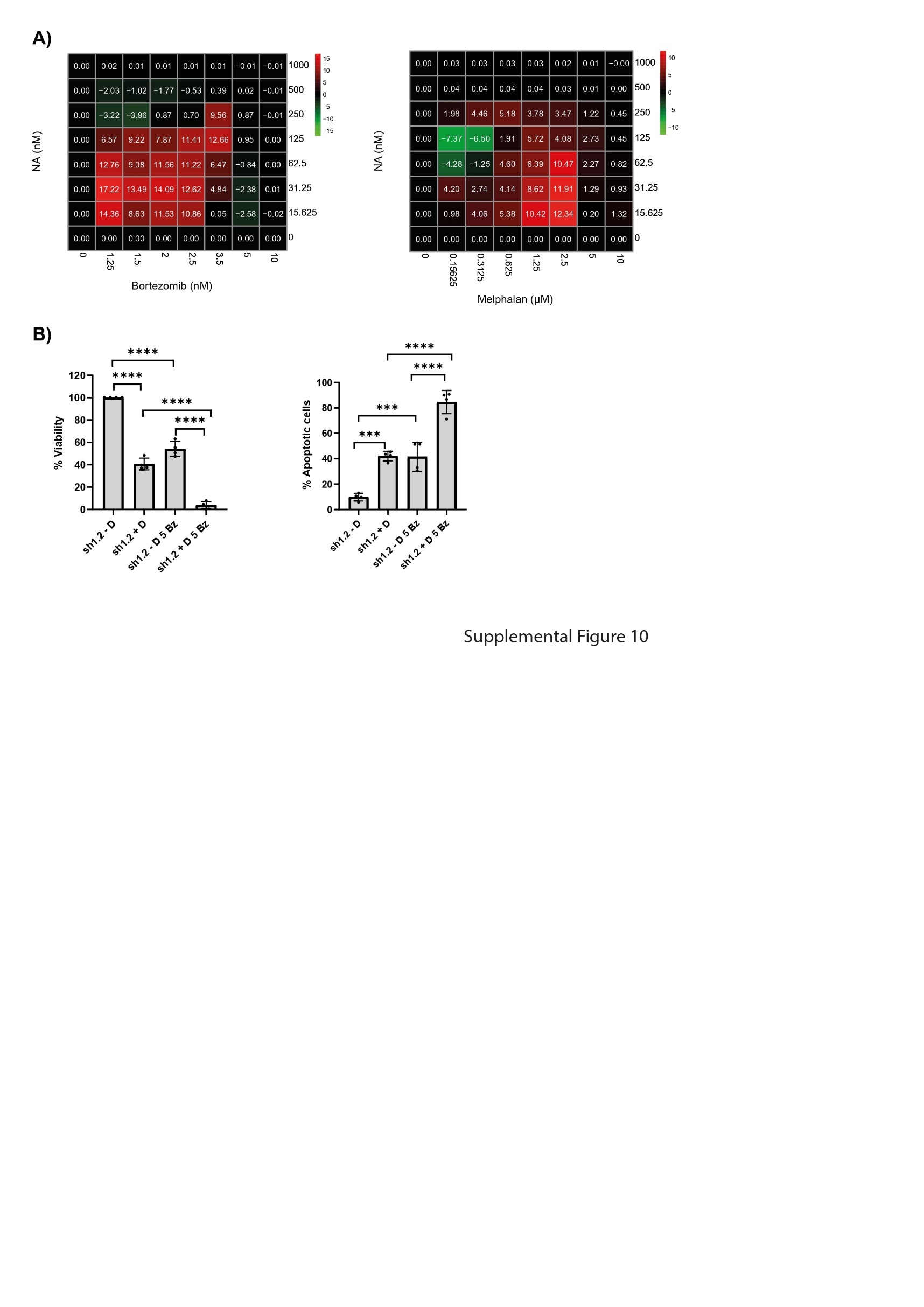


**Supplemental Figure 10:** **Nanaomycin A treatment sensitizes myeloma cells to the standard of care agents bortezomib and melphalan. A)** Effect of NA on bortezomib (Bz)- or melphalan (Mel)-induced cell death for AMO-1 cells. Cells were treated for 4 days with increasing doses of NA and/or Bz or Mel after which the viability was assessed using the CellTiter-Glo Luminescent Cell viability assay. Synergy scores were calculated using the Bliss method. The mean of at least three experiments is shown. **B)** Effect of DNMT3B depletion on the Bz response. shDNMT3B XG-2 cells were treated with doxycycline for three days followed by a treatment with Bz (5 nM) for 24h and the effect on viability and apoptosis were evaluated using the CellTiter-Glo Luminescent Cell viability assay and AnnexinV/7’AAD stainings followed by flow cytometric analysis respectively. The % apoptotic cells are the sum of AnnexinV (+) and AnnexinV (+)/7’AAD (+) cells. *** Indicates p≤0.001 and **** Indicates p≤0.0001.

**
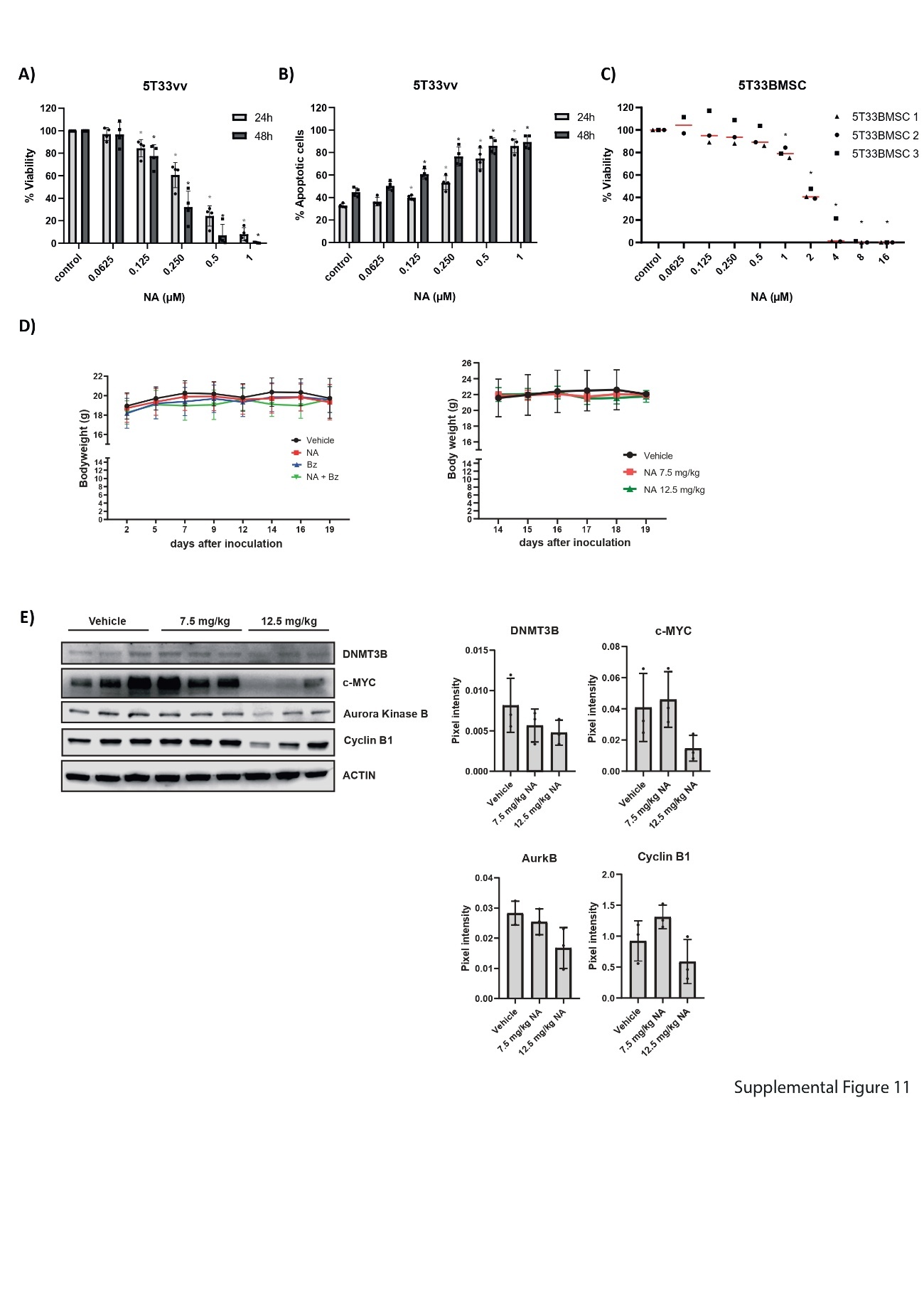
**

**Supplemental Figure 11: Effect of Nanaomycin A treatment on murine 5T33vv cells, 5T33MM derived BMSC and 5T33MM mice.** **A-B)** Effect of NA on the cell viability (A) and apoptosis (B) of 5T33vv cells. Cells were treated with indicated doses of NA treatment for 24h (light gray bars) and 48h (dark grey bars) and the effect on viability and apoptosis was evaluated using the CellTiter-Glo Luminescent Cell viability assay and AnnexinV/7’AAD stainings followed by flow cytometric analysis respectively. The % apoptotic cells are the sum of AnnexinV (+) and AnnexinV (+)/7’AAD (+) cells. **C)** Effect of NA on the cell viability of 5T33 derived BMSC. Cells were treated with indicated doses of NA for 4 days and the effect on viability was measured using the CellTiter-Glo Luminescent Cell viability assay. * Indicates p≤0.05 compared to control. **D)** Body weight of treated 5T33MM mice. Left: body weight monitored over time for mice treated with NA and/or Bz for three weeks. Right: body weight monitored over time for mice treated with NA for 5 consecutive days before reaching end-stage disease. **E)** Western blot analysis of the protein levels of DNMT3B and related targets in the tumor cells retrieved from the 5T33MM mice following treatment with NA for 5 consecutive days. Left: observed expression of the indicated proteins for each individual mouse is shown. Actin was used as loading control. Right: Pixel intensity of the protein levels relative to actin as measured by Image Studio.


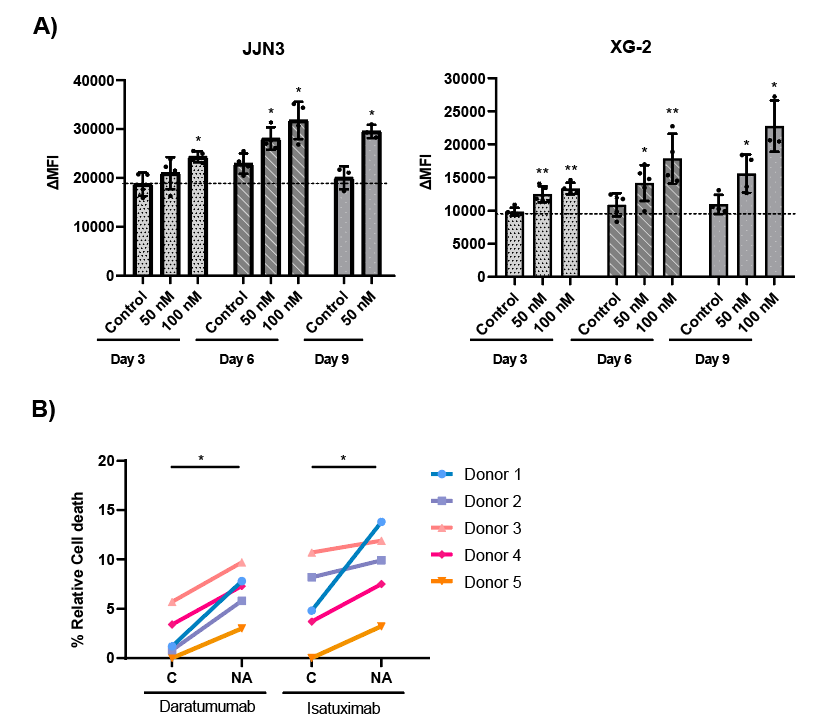


**Supplemental Figure 12:** **Nanaomycin A treatment sensitizes myeloma cells to the CD38 monoclonal antibodies daratumumab and isatuximab.** **A)** Effect of long-term low dose NA treatment (up to 9 days) on CD38 cell surface expression for the JJN3 and XG-2 cell lines. * Indicates p≤0.05 compared to control. **B)** Effect of long term NA treatment (75 nM, 9 days) on ADCC induced by daratumumab (1 µg/mL) and isatuximab (10 ng/mL) treatment for JJN3 cells. NK cells were added in effector-to-target ratio of 5:1. * Indicates p≤0.05 compared to C (untreated cells).

**Supplemental Tables**

| *Clone IDs* | | | | | |
| --- | --- | --- | --- | --- | --- |
| Name | **Catalog number** | **Source clone ID** | **Vector** | **Gene symbol or microRNA ID** | **Gene target sequence** |
| *Construct 1* | V3SH7669-225310009 | V3IHSMCG_5247659 | piSMART mCMV/TurboGFP | *DNMT3B* | GCCGGGATCGCTTCCTTGA |
| *Construct 2* | V3SH7669-227039440 | V3IHSMCG_6977090 | piSMART mCMV/TurboGFP | *DNMT3B* | CTAGGGTGCGAGCTGGAA |
| *Construct 3* | V3SH7669-230374420 | V3IHSMCG_10312070 | piSMART mCMV/TurboGFP | *DNMT3B* | CCAGTGACACGGGGCTTGA |

**Supplemental Table 1:** Clone IDs of the different virus constructs.

**Supplemental Table 2:** Genes deregulated upon *DNMT3B* knockdown.

**Supplemental Table 3:** Enriched hallmark pathways in the deregulated genes upon *DNMT3B* knockdown.

**Supplemental Table 4:** Enriched C2 pathways in the deregulated genes upon *DNMT3B* knockdown.

|  | **Age** | **Gender** | **Status** | **Heavy/light chain** | **t(4;14)** | **t(11;14)** |
| --- | --- | --- | --- | --- | --- | --- |
| **Patient 1** | 80 | M | Relapse | IgA Lambda | No | No |
| **Patient 2** | 70 | F | Relapse | Lambda | No | No |
| **Patient 3** | 68 | M | Relapse | IgG Lambda | No | No |
| **Patient 4** | 60 | F | Relapse | IgM Kappa | No | No |
| **Patient 5** | 69 | M | Diagnosis | IgA Lambda | No | No |
| **Patient 6** | 83 | F | Relapse | Lambda FLC | No | No |
| **Patient 7** | 72 | F | Relapse | IgG Lambda | No | No |
| **Patient 8** | 58 | M | SMM | IgA Lambda | No | Yes |
| **Patient 9** | 58 | M | Stable disease, no relapse | Kappa FLC | No | No |

**Supplemental Table 5:** Patient characteristics including age, gender (M: male and F: female), status, heavy/light chain and presence or absence of t(4;14) and/or t(11;14) translocations.

**References**

1. Zhan, F. *et al.* The molecular classification of multiple myeloma. *Blood* **108**, 2020-2028 (2006).

2. Vikova, V. *et al.* Comprehensive characterization of the mutational landscape in multiple myeloma cell lines reveals potential drivers and pathways associated with tumor progression and drug resistance. *Theranostics* **9**, 540-553 (2019).

3. Tessoulin, B. *et al.* Whole-exon sequencing of human myeloma cell lines shows mutations related to myeloma patients at relapse with major hits in the DNA regulation and repair pathways. *Journal of Hematology & Oncology* **11** (2018).

4. Alaterre, E. *et al.* Comprehensive characterization of the epigenetic landscape in Multiple Myeloma. *Theranostics* **12**, 1715-1729 (2022).

5. Moreaux, J. *et al.* A high-risk signature for patients with multiple myeloma established from the molecular classification of human myeloma cell lines. *Haematologica-the Hematology Journal* **96**, 574-582 (2011).

6. De Smedt, E. *et al.* G9a/GLP targeting in MM promotes autophagy-associated apoptosis and boosts proteasome inhibitor-mediated cell death. *Blood Adv* **5**, 2325-2338 (2021).
